# AGEs and RAGE: metabolic and molecular signatures of the glycation-inflammation axis in malignant or metastatic cancers

**DOI:** 10.37349/etat.2023.00170

**Published:** 2023-09-28

**Authors:** Gowri Palanissami, Solomon F.D. Paul

**Affiliations:** University of Toronto, Toronto, Canada; Department of Human Genetics, Faculty of Biomedical Sciences and Technology, Sri Ramachandra Institute of Higher Education and Research (Deemed to be University), Porur, Chennai 600 116, Tamil Nadu, India

**Keywords:** RAGE, AGEs, high mobility group box-1, S100, cancer, glycation, epigenome, microbiome

## Abstract

From attributing mutations to cancers with the advent of cutting-edge genetic technology in recent decades, to re-searching the age-old theory of intrinsic metabolic shift of cancers (Warburg’s glycolysis), the quest for a precise panacea for mainly the metastatic cancers, remains incessant. This review delineates the advanced glycation end product (AGE)-receptor for AGE (RAGE) pathway driven intricate oncogenic cues, budding from the metabolic (glycolytic) reliance of tumour cells, branching into metastatic emergence of malignancies. Strong AGE-RAGE concomitance in metastasis, chemo-resistance and cancer resurgence adversely incite disease progression and patient mortality. At the conjunction of metabolic and metastatic shift of cancers, are the “glycolytically” generated AGEs and AGE-activated RAGE, instigating aberrant molecular pathways, culminating in aggressive malignancies. AGEs as by-products of metabolic insurgence, modify the metabolome, epigenome and microbiome, besides coercing the inter-, intra- and extra-cellular micro-milieu conducive for oncogenic events like epithelial-mesenchymal transition (EMT). AGE-RAGE synergistically elicit ATP surge for surplus energy, autophagy for apoptotic evasion and chemo-resistance, insulin-like growth factor 1 (IGF-1) for meta-inflammation and angiogenesis, high mobility group box-1 (HMGB1) for immune tolerance, S100 proteins for metastasis, and p53 protein attenuation for tumour suppression. AGEs are pronouncedly reported in invasive forms of breast, prostate, colon and pancreatic cancers, higher in patients with cancer than healthy counterparts, and higher in advanced stage than localized phase. Hence, the investigation of person-specific presence of AGEs, soluble RAGE and AGE-activated RAGE can be advocated as impending bio-markers for diagnostic, prognostic and therapeutic purposes, to predict cancer risk in patients with diabetes, obesity, metabolic syndrome as well as general population, to monitor prognosis and metastasis in patients with cancer, and to reckon complications in cancer survivors. Furthermore, clinical reports of exogenous (dietary) and endogenous (internally formed) AGEs in cancer patients, and contemporary clinical trials involving AGE-RAGE axis in cancer are underlined with theranostic implications.

## Introduction

Persistent metabolic burden based oxidative stress in physiological ageing, and chronic hyperglycemic abundance derived glycative stress in diabetes, cancers and concomitant diseases expose both intra- and extra-cellular biomolecules to glycoxidative insults [1–3]. Non-enzymatic glycation of biomolecules commences with the cross-linking of lysine, arginine or cysteine-amino groups of proteins, lipids or nucleic acids, non-enzymatically to carbonyl groups of reducing sugars/glycolytic intermediates. This Maillard reaction includes a sequel of events entailing unstable Schiff’s bases and Amadori products, further undergoing biochemical alterations to elicit glycated adducts called advanced glycation end products (AGEs) [4]. Hyperglycemia and oxidative stress play a vital role in the generation of AGEs. AGEs and reactive oxygen species (ROS) elicit DNA adducts, DNA strand breaks, mutagenesis causing genomic instability, and act as indispensable factors in bringing about ageing related pathological changes at both cellular and molecular levels [5]. Glycation promotes inflammation and oxidative stress, enhancing ageing associated bio-molecular insults [6]. Many studies have reported the involvement of glycated proteins (Table 1) in multiple degenerative and inflammatory diseases: glycation of cerebro-spinal fluid (CSF) proteins in Alzheimer’s disease; glycation of low-density lipoprotein (LDL), collagen and elastin in diabetic arteriosclerosis leading to cerebrovascular and cardiovascular occlusion; glycation of lens crystallins in diabetic cataract; elevated carboxymethyl lysine in CSF of amyotropic lateral sclerosis (ALS); presence of argpyrimidine and carboxymethyl lysine in cancer tissues; with implications of glycated high-density lipoprotein (HDL) in diabetes and cancer; glucose, glyceraldehyde and methyl glyoxal AGEs in cancer proliferation and invasion [4, 7–13]. Protein glycation promotes the conversion of α-helix to β-sheet structure reported in β-amyloid protein of Alzheimer’s and β-synuclein of Parkinson’s diseases [14].

**Table 1:** List of AGEs studied in cancer so far

**Categories of AGEs**	**AGE modifications identified**	**References**
AGE-modified proteins/DNA	Annexin II Apolipoprotein B100 N^2^-(1-carboxyethyl)-2’-deoxyguanosine (CEdG)-DNA adducts Collagen type IV Crystallin DNA- and RNA-binding proteins DNA-histone crosslinks Fibrinogen Glycerol-3-phosphate dehydrogenase (G3PDH) HDL Glycated haemoglobin (HbA1C) Heat shock proteins (HSPs) Human serum albumin (HSA) Histone proteins (H1, H2A and H2B) Lactate dehydrogenase (LDH) Prohibitin Transcription factors Splicing factors	[15–21]
Low molecular structures produced during glycation	Argpyrimidine Fructosylysine Imidazole Methylglyoxal (MG) lysine dimer Nε carboxy-methyl lysine (CML) Nε carboxy-ethyl lysine (CEL) Nδ-(5-hydro-5-methyl-4-imidazolon-2-yl)-ornithine (MG-H1) Pyrraline Pentosidine	[16, 19, 22, 23]
Unspecified structures formed by alterations with certain glycating agents	Acetaldehyde-derived AGEs Glucose-derived AGEs Glyceraldehyde-derived AGEs Glycoaldehyde-derived AGEs Glyoxal-derived AGEs MG-derived AGEs 3-Deoxyglucosone-derived AGEs	[19, 24–26]

MG-H1: MG-derived hydroimidazolone

**Figure 1:**
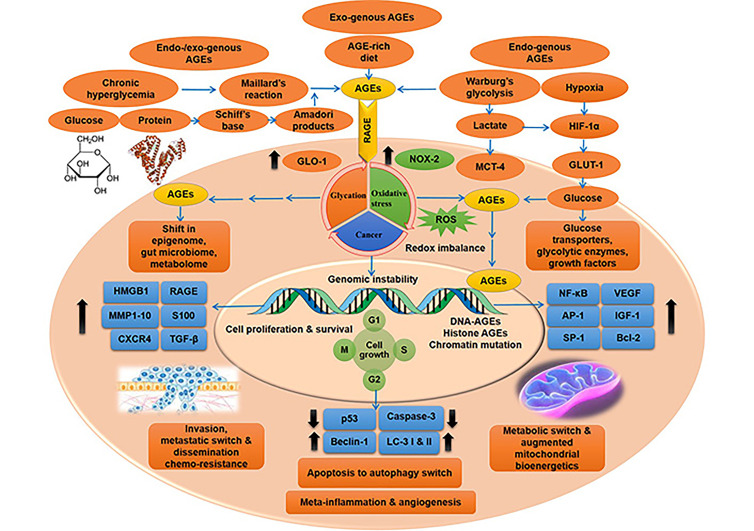
AGE-RAGE pathway driven intricate molecular cues in cancer cells, giving rise to metastasis and chemo-resistance. Oncogenic cues triggered by metabolic imprint of AGEs and up-regulated glycolytic reactions, under conditions of hyperglycemia and hypoxia (also Warburg’s aerobic glycolysis), respectively, with the mutagenic exposure of exogenous (dietary) and endogenous AGEs, culminating in activated RAGE-driven metabolic, apoptosis to autophagy and metastatic switch, characteristic of malignant transformation and capable of eliciting chemo-resistance. Multi-stage transformation of metabolic switch of cancer cells to metastatic surge, driven by AGEs and RAGE-mediated aberrant molecular cues, result in the concurrent generation of multiple hallmarks of cancer, from altered mitochondrial bio-energetics to sustained proliferative signals, redox imbalance, evasion of tumour suppression, apoptotic evasion, inflammation, angiogenesis, invasion and metastasis. HIF-1α: hypoxia-inducible factor 1α; GLO-1: glyoxalase-1; NOX-2: reduced nicotinamide adenine dinucleotide phosphate (NADPH) oxidase enzyme-2; MCT-4: monocarboxylate transporter-4; GLUT-1: glucose transporter-1; MMP1: matrix metalloproteinase 1; CXCR4: C-X-C chemokine receptor 4; TGF-β: transforming growth factor β; LC-3: microtubule-associated protein light chain 3; VEGF: vascular endothelial growth factor; SP-1: stimulator protein-1; Bcl-2: B cell lymphoma protein-2; G1: first gap or growth phase; M: mitosis phase; S: synthesis phase

**Figure 2:**
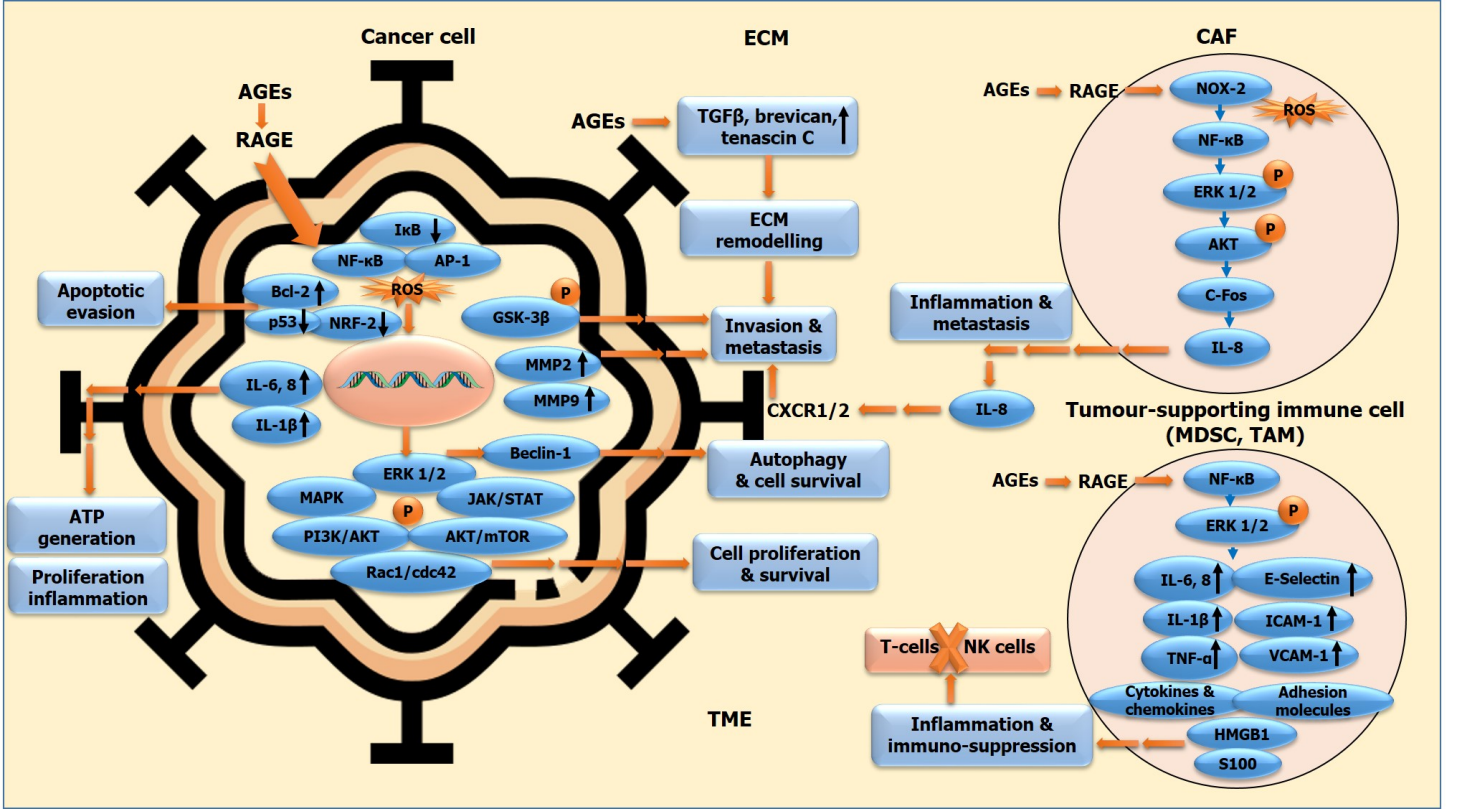
AGE-RAGE signalling cascade incited in different cellular components of tumour-micro milieu facilitating malignant transformation. Oncogenic molecular cues in cancer cells, and in stromal component, comprising of CAFs, MDSCs, TAMs and other tumour-supporting immune cells, all bound together by ECM, constituting the tumour micro-environment (TME). The molecular interplay between cancer cells and associated stromal cells via AGE-RAGE axis generates a TME conducive for the elicitation of malignant features of cancer, via incitation of cell proliferation and survival, evasion of apoptosis and tumour suppression, autophagy induction and chemo-resistance, tumour-promoting inflammation and immune suppression, ultimately leading to ECM remodelling, invasion and metastasis. IκB: NF-κB inhibitor; GSK-3β: glycogen synthase kinase 3β; c-Fos: FBJ murine osteosarcoma viral oncogene homolog (AP-1 transcription factor subunit); MDSC: myeloid-derived suppressor cell; TAM: tumor associated macrophage; P: phosphorylation

Receptor for AGE (RAGE) is a 45-kDa cell surface, membrane bound receptor, composed of three extracellular domains—V, C1 and C2; a trans-membrane helix; and a short cytoplasmic tail. This pattern recognition receptor (PRR) forms a member of immunoglobulin (Ig) superfamily, capable of binding a broad repertoire of ligands mainly, AGEs, damage-associated molecular patterns (DAMPs)- high mobility group box-1 (HMGB1) and S100 (calgranulin/calcium binding) group of proteins, with significant oncogenic role. RAGE also binds more ligands—β2 integrin/macrophage-1 (MAC-1) antigen, amyloid β peptide and so on [27]. During embryonic development, RAGE displays high expression, mainly in brain, but its expression declines in adult tissues. Many cells express RAGE, particularly under conditions of pathological and inflammatory stress, including smooth muscle cells, nerve cells, endothelial cells, neutrophils, MACs, mast cells activated immune cells and cancer cells. *RAGE* gene is located on chromosome 6, in the major histocompatibility complex (MHC) class III region of the MHC, associated with immune responses. RAGE cytosolic domain is devoid of intrinsic tyrosine kinase activity or similar motifs for executing downstream effector signals. Instead, the trans-membrane receptor binds with Diaphanous-1 (Dia-1), which is a member of formin protein family and serves to regulate cell motility, actin cytoskeleton reorganisation and elicits signalling via transcription factors [28]. RAGE expression is prompted by AGEs and its other induction factors include—high glucose, hypoxia, ROS and pro-inflammatory mediators. AGE-RAGE interaction stimulates a vast array of cell signalling pathways and transduction factors, as shown in Figures 1 and 2 [29, 30]:

(1) Signalling pathways—mitogen-activated protein kinase (MAPK), extracellular signal-regulated kinase 1/2 (ERK 1/2), p38, c-Jun N-terminal kinase (JNK), phosphatidylinositol 3-kinase (PI3K).
(2) Transcription factors—nuclear factor kappa B (NF-κB), activating protein-1 (AP-1), signal transducer and activator of transcription 3 (STAT-3).
(3) Oncogenic rat sarcoma protein (Ras), protein kinase C (PKC).
(4) Members of Ras homolog (Rho)/guanosine triphosphate binding hydrolase enzymes (GTPase)-cell division control protein 42 (cdc42) and Ras-related C3 botulinum toxin substrate 1 (Rac1) signalling pathways.
(5) ROS generation.

Altogether they promote the multiple stages of tumour growth to surge metastasis by facilitating cell survival and hyper-proliferation, migration and invasion, release of growth factors and pro-inflammatory cytokines, and motility with alterations in cell shape. In this review, the AGE-RAGE pathway driven facilitation of intricate oncogenic cues in tumour-micro environment, budding from the metabolic (glycolytic) reliance of tumour cells triggering superfluity of AGEs, branching into metastatic emergence of malignancies via RAGE-reliant and -independent mechanisms, with alterations in epigenome, gut microbiome and metabolome. are delineated in detail. Further, the elucidation of AGEs, RAGE and soluble RAGE (sRAGE) in patient samples and experimental models, along with various research studies including clinical trials on the diagnostic and therapeutic evaluation of AGE/RAGE inhibitors in different cancers have been elaborately discussed.

## Metabolic reliance and metastatic shift of cancer cells

Unrestricted cell growth, as a vital hallmark of cancers, derives energy for increasing its bio-mass by boosting nutrient supply via rewired metabolic pathways of cancer, generating the metabolic reliance of cancer cells. This metabolic re-modelling to augment cancer growth and cell cycle progress, has been related to one of the most characteristic features of cancer cells-cell migration.

Differences in the metabolic status of cancer cells are the determinants of their metastatic ability [31]. It accentuates that metabolic distinction between cancers and their metastatic propensity are inter-reliant. Hence recognising novel metabolic reliance of cancer cells subsidising the multi-stage evolution of cancer hallmarks could provide potential therapeutic targets for successful cancer treatment.

High propensity for metastasis and poor survival in cancer-afflicted patients with diabetes than the non-diabetic counterparts, considering their exposure to long-term high glucose and hence AGEs, cites the powerful interplay of AGEs in the invasiveness and hence malignancy of cancers, via both RAGE-dependent and independent means. AGEs arising as a result of glycolytic superfluity, especially in cancer cells and concomitant micro milieu undergoing Warburg’s type of metabolism (glycolysis), confer highly proliferative and metastatic phenotype to these cells via RAGE activation, making them biologically aggressive in par with their atypical metabolic activity (shown in Figure 1). Age-related alterations in extracellular matrix (ECM) regulate cancer cell invasion and metastasis [31], and AGEs wreak damage to ECM components by age-pertinent accretion of irreparable adducts of macro-molecules [32]. Abnormal ECM promotes oncogenic transformation and metastasis. AGE mediated modification of long-lived matrix components—collagen, fibronectin and laminin pave way for cancer promotion and invasiveness, possibly via RAGE activation. ECM stiffness caused by non-enzymatic glycation of matrix proteins play active role in tumour progression and alteration of the tumour response to therapy. Age-concomitant tissue-level modifications of brain meninges by exposure to glycated collagen *in vitro*, reveal the potentiality of AGEs to elicit glycation-driven structural and functional changes in ECM/meningeal membrane of brain [33]. AGEs/RAGE/HIF-1α axis augments glycolysis; constitutes a crucial signalling axis influencing glucose metabolism [12].

Metastatic events primarily require the cellular and molecular interplay between cancer cells and their micro milieu components, which is strongly facilitated by RAGE. The epithelial-mesenchymal (EM) transition phenomena confer metastatic characteristics to cancer cells, by promoting motility, invasion and apoptotic evasion, which all are indeed shown to be augmented by RAGE in cancer cells [12].

Role of RAGE in subsidising mitochondrial bio-energetics (ATP) and metastatic dissemination EMT are to be underlined, considering the AGEs-RAGE interlink and the upregulated glycolytic rate in cancer cells (shown in Figure 1), and hence lactate turnover, the main fuel for metastatic cancers [31, 34].

## Metabolic imprint of AGEs in cancers

Manifestation of malignant features or malignant nature of cancer cells indispensably demands the alteration in metabolic status of cancer cells, which has been confirmed consistently, by as early as Otto Warburg to the most recent scientific studies of cancer metabolic phenotype. The basic build-up of cancer cells stipulating surplus energy (ATP) plus multiple micro- and macro-nutrients, is met with by glycolytic superfluity, ensuing AGEs accretion and lactate abundance, the main energy source of cancer cells and facilitator of metastasis. Molecular interplay between metabolic network cues are highly complex and are also involved in multiple cellular processes, both physiological and pathological, hence becoming hard to target and harder to treat. This is where the AGEs and its receptor RAGE come into play, acting as the major upstream or downstream converging point of myriad pathogenic events responsible for developing and disseminating cancers. As revealed by Krisanits and colleagues [35], AGEs leave an indelible mark in the normal breast tissues, with the metabolic imprint of AGEs developing into potential hyperplastic lesions via molecular initiation of RAGE. This “metabolic memory” created by AGEs in otherwise normal tissue micro-milieu is indispensable for the impending instigation of cancers [36–38].

Exogenous AGE treatment of prostate cancer cells *in vivo* incited RAGE dimerization in stimulated fibroblasts/stromal cells, resulting in sustenance and augmentation of migratory capacity of cancer cells. However, RAGE depletion in stromal cells arrested the AGE-driven cancer cell growth. This implies the role of AGEs-RAGE in the crosstalk between tumour cell and tumour-associated fibroblasts/stroma in eliciting cancer progression and metastasis, and the role of AGE-rich diet in inciting tumorigenic events via molecular interplay of tumour micro-milieu [39].

Critical events concomitant with altered biochemical niche in mammary glands during normal developmental phase, greatly impact the cellular programs in breast micro-milieu. Exposure to AGEs leave a long-lasting imprint on the cellular microenvironment similar to metabolic memory in diabetic patients [37]. The longevity of AGEs in tissues, coupled with their pro-inflammatory and pro-oxidant characteristics contribute to their long standing pathogenic effects in various diseases including cancer and diabetes. High AGE-rich dietary consumption, especially in pubertal age, elicits breast cancer risk via substantial dysregulation in normal growth of mammary glands and promotion of hyperplastic lesions by adulthood [35]. This induction of hyperplastic lesions remains irreversible even with diet intervention or diet reversal i.e. follow up with non-AGE/control diet. AGEs leave a “metabolic imprint” in the normal mammary gland micro-milieu, with everlasting biological changes characteristic of futuristic breast cancer development, via both RAGE-dependent and independent mechanisms [38]. Permanence of hyperplastic lesions despite diet intervention, calls for the dire need to derive an effective therapeutic interception of AGEs with implications in prevention of cancer incidence as well as cancer progression and invasion [36].

AGE driven intensification in cancer concomitant phenomena are reliant on RAGE. Anomalous AGE accretion potentially indicate a metabolic pre-disposition to cancer development [40]. Metabolites in cancer form a major object of study, and AGEs as highly reactive metabolites in cancer serve as potential targets for early and effective treatment besides early detection of cancers (as seen in Table 2).

**Table 2:** Recent studies evaluating the pathogenic and predictive role of AGEs in cancer

Investigation of AGEs	Involvement of AGEs in cancers	References
**In patient samples (cancer/control)**		
Analysis of genetically modified circulating levels of AGEs in 1,051 patients with breast cancer by ELISA	Higher levels of AGEs and AGEs/sRAGE-ratio—correlated with increased breast cancer risk sRAGE levels—negative correlation with breast cancer risk AGEs—positive correlation with bad cancer prognosis	[41]
Analysis of plasma AGEs in 1,378 patients with primary colorectal cancer by ultra-performance liquid chromatography-tandem mass spectrometry	Higher ratio of MG-derived AGEs *versus* those derived from glyoxal displayed a strong positive correlation with colorectal cancer risk CML, CEL and MG-H1—inverse correlation with colorectal cancer risk	[23]
Analysis of dietary AGEs intake in 450,111 participants by European Prospective Investigation into Cancer and Nutrition (EPIC) study	CML and MG-H1—inverse correlation with colorectal cancer risk, but not for CEL AGEs analysed in this study may not be colorectal cancer-promotive	[42]
Analysis of dietary AGEs intake—prospective observational study by Women’s Health Initiative (WHI, WHI-USA) in 2,073 women with invasive breast cancer	After a median 15.1 years of follow-up, 642 deaths were registered, including 198 breast cancer specific and 129 cardio-vascular specific deaths Higher consumption of dietary AGEs (CML-AGEs) after cancer diagnosis in post-menopausal women—correlated with enhanced risk of mortality from cancer as well as cardio-vascular diseases	[43]
Analysis of dietary AGEs intake—Prostate, Lung, Colorectal and Ovarian (PLCO) Cancer Screening Trial in cancer-free healthy women	After a median 11.5 years of follow-up, 1,592 women were diagnosed with breast cancer in the PLCO Highest CML-AGE intake—related to increased breast cancer risk (*in situ* and hormone receptor positive breast cancer) and mortality risk in healthy women Increased CML-AGE intake post-diagnosis coupled with lower intake of fruits and vegetables—associated with increased mortality rate in both hormone receptor positive and negative breast cancers	[44]
Analysis of plasma AGEs—a 5-year follow-up cohort study by Shaanxi Provincial People’s Hospital, in 131 patients with stage II and III breast cancer, surgically treated	Incidence of metastasis significantly associated with serum AGE concentrations in the patients For the period of follow-up, metastasis interval was shorter in diabetic than non-diabetic subjects	[45]
Analysis of plasma AGEs in peripheral blood mononuclear cells (PBMCs) from 20 adult survivors of paediatric Hodgkin’s lymphoma (HL)	Plasma AGEs (CML and MG-H1)—significantly higher in HL survivors than healthy subjects Higher levels of RAGE, NADPH oxidase, oxidative stress, NF-κB and IL-6 expression, along with weakened anti-oxidant defence Co-existence of AGEs, oxidative and inflammatory stress in HL survivors Potentially detrimental in initiating long-term complications in HL survivors	[46]
Analysis of MG AGEs/adducts in blood-derived cultures (BDCs, liquid biopsy) of patients with cancer (18 localized and 20 advanced cases)	Relatively higher levels of MG glycated adducts reported in tumour tissues than the normal counterparts Higher MG adducts (MGAs) in advanced cancers than the ones in localized phase	[26]
Analysis of cutaneous AGEs by skin auto-fluorescence (SAF) in type-2 diabetic patients	SAF is indicative of pentosidine, an AGE prevalent in skin biopsies SAF values > 2.6 projected a 2.6 fold increased risk of cancer with significant association between AGEs and cancer incidence Diabetic patients who had cancer or went onto develop new cancers had considerably higher initial SAF values than those who did not have or develop cancer	[47]
Analysis of histone-glycation adducts in SKBR3 breast cancer cells *in vitro*, MCF7 and CAMA-1 tumor xenografts *in vivo* and tumour samples from patients with metastasis or recurrence	Histones are basally glycated in breast cancer Histone glycation disrupts chromatin architecture, nucleosome assembly and stability Breast cancer cells, xenografts, as well as patients’ tumours showed high basal histone glycation levels and deglycase enzyme (DJ-1) overexpression Link between metabolic perturbation and epigenetic dysregulation in cancer	[48]
**In animal (mice) models—*in vivo***		
Analysis of impact of dietary AGEs in pubertal FVB/n mice (model of breast cancer), fed a high AGE diet	High AGE-rich dietary consumption, especially in pubertal age, elicit breast cancer risk via substantial dysregulation in normal growth of mammary glands and promotion of hyperplastic lesions by adulthood AGEs leave a “metabolic imprint” in the normal mammary gland micro-milieu, with potential risk for breast cancer development	[37]
Analysis of impact of dietary AGEs in wild type FVB/n and RAGE null (RAGE^–/–^) mice, fed persistent high AGE diet (chronic dietary-AGE model of breast cancer)	By influencing cellular matrix, AGEs perturb developmental programs during puberty and induce breast cancer growth AGE driven changes in tissue architecture and cell function led to 3-fold rise in neoplastic growth Both RAGE-dependent and independent mechanisms were involved in eliciting the same Dietary AGE activated RAGE-stimulated stroma resulting in pre-neoplastic lesions, continuing into adulthood	[38]
Analysis of impact of dietary AGEs in FVB-RAGE^+/+^ and FVB-RAGE^–/–^ xenograft mice (prostate cancer model), fed AGE-rich diet	AGE treatment prompted RAGE dimerization in activated fibroblasts AGEs elicited RAGE-mediated sustenance and augmentation of migratory capacity of cancer cells RAGE depletion in tumour stroma blocked AGE-driven cancer expansion	[39]
**In cancer cells—*in vitro***		
Analysis of glycation in MCF-7 cells by mass spectrometry	Revealed 17 glycated sites, majority in arginine residues and functional domains	[49]
Analysis of proteins in liver cancer cells by mass spectrometry	Fructosamine-3-kinase (FN3K) sensitive glycation of 110 proteins—transcription factors, splicing factors, histones, DNA- and RNA-binding proteins, HSPs; HSP90AA1, HSP90AA4, translation factors (eIF4A1, eIF1, eIF3G), transcription factors (NRF-2), replication and repair proteins (HELB, MCM3), splicing factors (SRSF7, PUF60) and more importantly, enzymes involved in glucose metabolism (LDHA, LDHC), were identified	[17]

ELISA: enzyme-linked immunosorbent assay; eIF4A1: eukaryotic initiation factor 4A-1; NRF-2: nuclear factor erythroid 2-related factor 2; HELB: DNA helicase B; MCM3: minichromosome maintenance complex component 3; SRSF7: serine/arginine-rich splicing factor 7; PUF60: Poly(U)-binding-splicing factor; LDHA: lactate dehydrogenase A; LDHC: lactate dehydrogenase C; IL-6: interleukin-6

In a prospective observational study conducted by the WHI-USA involving 2,073 women diagnosed with invasive breast cancer, higher consumption of dietary AGEs (CML-AGEs) after cancer diagnosis in post-menopausal women, correlated with their enhanced risk of mortality from cancer as well as cardio-vascular diseases [43]. High ingestion of dietary AGEs (CML-AGEs) enhanced breast cancer risk in healthy women, and higher dietary AGEs coupled with lower intake of fruits and vegetables in women post-cancer diagnosis were associated with increased mortality rate in both hormone receptor positive and negative breast cancers [44]. The indispensable yet hidden role of beneficial dietary phytochemicals, especially flavonoids and other polyphenols, present in fruits and vegetables, cannot be underestimated with regard to their promising anti-oxidant effects and potential insinuations in curbing AGEs. Higher than normal plasma AGE levels with concomitant augmentation of RAGE expression, oxidative stress and inflammatory markers (potential contributors of pre-mature aging), were reported recently in PBMCs isolated from adult survivors of paediatric HL, with potentially detrimental implications in initiating long-term complications in those survivors [46].

Hence the metabolic imprint of AGEs in cancer instigation and metabolic insurgence of AGEs in cancer aggression via upregulated RAGE signalling cascade, coupled with oxidative and inflammatory stress, are to be elaborately studied as pertinent bio-markers for precise diagnostics and potential targets for personalized therapeutics.

## Metabolic insurgence and cancer malignance

Molecular interplay between cancer cells and the concomitant stromal components in the tissue micro-milieu are vital in the propagation of cancer via various factors like EMT, promotion of cell motility and invasion. Cancer cells undergo remodelling of their metabolic niche to cope with the upcoming surplus energy demands for synthesis of new molecules and sustenance of oncogenic milieu. Up-regulated glycolytic events partake in meeting the unrestrained energy requirements of cancer cells. Higher the glycolytic rate, greater is the biologically aggressive behaviour of cancers. With unlimited glycolytic by-products generated amounting up to augmented AGEs and lactate levels, glycolytic upsurge directly points to the rise in metastatic potential of cancer cells, by means of AGEs-driven RAGE signalling, and lactate turnover, the prime fuel for metastatic cancers (as shown in Figure 1).

Correlation between the metabolic phenotype and molecular genotype of cancers are quite significant in ruling out the malignant behaviour of cancers. Metabolic interaction between tumor cells and the stromal cells varies in different cancers, giving rise to differences in their aggressive nature. Based on the analysis of glycolytic status of tumours, by evaluating the immunohistochemical expression of GLUT-1 and carbonic anhydrase IX (CAIX) in tissue microarray sections from 740 patients with breast cancer, Choi and colleagues [50] classified the molecular subtypes of breast cancer into the following metabolic strata:

(1) Warburg type: tumor—glycolysis type, stroma—nonglycolysis type.
(2) Reverse Warburg type: tumor—nonglycolysis type, stroma—glycolysis type.
(3) Mixed type: tumor—glycolysis type, stroma—glycolysis type.
(4) Null type: tumor—nonglycolysis type, stroma—nonglycolysis type.

Warburg effect theory designates “the metabolic shift from mitochondrial oxidative phosphorylation (OXPHOS) to glycolysis in tumours; glycolysis is the major metabolic process in tumour cells”. Reverse Warburg effect theory describes the glycolysis in stromal cells with the glycolytic end products eliciting “tumor cell growth and survival through efficient generation of ATP by OXPHOS in the mitochondria”.

Triple negative breast cancer (TNBC) constitute the majority of Warburg type, where the tumour cells follow glycolytic metabolism and the mixed type, where both the tumour and stromal cells display glycolytic metabolism, indicating metabolically active and biologically aggressive tumours. On the contrary, luminal breast cancers display reverse Warburg type, where only the stromal cells follow glycolytic metabolism and null type, where the tumour as well as stromal cells do not follow glycolytic metabolism, indicating metabolically inactive and biologically nonaggressive tumours.

Expression of metabolism- and autophagy-related proteins such as GLUT-1, MCT-4, ATP synthase, LC-3I and LC-3II, along with chemo-resistance and cancer relapse were noted the highest in TNBC, showing Warburg’s and mixed type of glycolysis, and the lowest in luminal A and B cancers, displaying reverse Warburg’s and null type of glycolysis. GLUT-1 positivity and MCT-4 were associated with high histologic grade and poor disease-free survival in patients. The high expression of metabolism- and autophagy-related proteins (GLUT-1, MCT-4, ATP synthase, LC-3I and LC-3II) have been linked to the development of metastasis and drug resistance in cancers. MCT-4, specifically expressed in cells undergoing high glycolytic rate, promotes lactate efflux.

The mixed type with both tumour and stromal cells undergoing Warburg’s glycolysis display higher histologic grade and Ki-67 (cellular marker for proliferation) index. But the null type with neither the tumour nor stromal cells undergoing glycolysis exhibit lower histologic grade and Ki-67 index. Luminal cancers demonstrate lower grade, mitotic index and lesser necrosis than the human epidermal growth factor receptor 2 (HER2) or TNBC forms, correlating less-active metabolic status of the tumor with less aggressive cancers. The Warburg and mixed types have explicit strong associations with highly invasive and resistant cancers like TNBC, while the reverse Warburg and null types relate with less aggressive forms like luminal breast cancer, signifying a link between the glycolytic status, metabolic phenotype and biology of cancers. Altogether, the glycolytic dependence of tumour cells relates directly with the biologically aggressive and malignant nature of cancers.

## Metabolic switch and metastatic impact of AGEs-RAGE

Metabolic insurgence or re-programming of cancers (dysregulated cellular bio-energetics) is strong and sufficient enough as a stand-alone hallmark capable of generating all the other hallmarks of cancer namely, sustained proliferative signals, tumour suppression, apoptotic evasion, inflammation, immune suppression, genomic instability, metastasis and angiogenesis via AGE-RAGE signalling cascade. Mitochondria are central to the maintenance of apoptotic and metabolic homeostasis. Present at the facade of eliciting chemo-resistance, mitochondria regulate molecular interplay at places of cellular stress and facilitate cell survival. Perturbed mitochondrial functions are commonly observed in cancer, often leading to altered nuclear gene expression via retrograde signalling and promotion of tumour endorsing stromal re-modelling [51]. Evasion of apoptosis and metabolic re-wiring are endorsed by the mitochondrial components invoking chemo-resistance and metastasis of cancers.

Metastasis and chemo-resistance, serve as key causative factors for mortality in patients with cancer, posing serious challenges and hindrances in their successful treatment. The typical conduit for discovering drugs, even for developing molecularly-targeted and immuno-therapy medicines, disregards the potential ability of trial drugs to impede metastasis [52]. RAGE upregulation triggers metastatic switch in (melanoma) cancer cells [53]. Enhanced glycolytic signature and exhaustive glucose uptake of cancer cells facilitate glycation reactions and the formation of AGEs (coupled with ROS generation), subsequently resulting in activation of RAGE and its other ligands—HMGB1 (redox-sensitive DAMP associated with inflammation and autophagy) and S100A4 (metastasis associated protein), with implications in cancer progression, metastasis and chemo-resistance.

Evasion of apoptosis and metabolic rewiring are suggested as two major execution pathways of complex drug resistance mechanisms mediating cancer cell survival, when exposed to cancer chemotherapy [54]. Besides facilitating metastasis and autophagy switch in cancer cells, RAGE along with HMGB1, has been shown to promote inflammatory pathway responsible for generating abundant ATP in the mitochondria of cancer cells, clearly establishing the role of RAGE in altered mitochondrial bio-energetics in neoplasms [34]. AGEs and RAGE have been implicated in the progression of thyroid cancer, the most common endocrine carcinoma, clinically challenging to treat due to its heterogeneous nature, with both genetic as well as non-genetic pre-disposition mainly cellular milieu conducive for carbonyl-, nitrosative- and oxidative stress [55].

Cancer cells re-model their metabolic phenotype and that of stromal cells in the surrounding micro-milieu to suit their surplus energy needs. With AGEs at the forefront of sustained glycolytic flux and concomitant ROS generation, incessant activation of RAGE incites its other ligands mainly S100A4, S100A7 and S100A8/A9 group of proteins pertinent to the derivation of pre-metastatic niche [56]. S100A4 takes part in the EMT, with considerably high expression in the invasive facade of cancers [18]. S100A4, related to aggressive metastatic phenotype, is a Wingless-related integration site (Wnt) target gene, which is positively regulated by Wnt/beta-catenin signalling. S100A4 also up-regulates (transcription factor) Snail and hence, suppresses E-cadherin expression, an important event in EMT activation [57, 58].

AGEs via RAGE predominantly elicit transmuted mitochondrial dynamics, higher oxidative load (ROS), mitigated apoptosis and augmented metastasis in cancer cells (as shown in Figure 1). AGE/RAGE-driven oxidative stress mediate bio-molecular damage and pave way for cell death in normal cells via apoptosis induction, while prevents apoptosis (anti-apoptotic) in cancer cells via autophagy induction, thereby prompting resistance and promoting metastasis [59].

AGEs exert their action via RAGE-driven surge in (1) the expression of various genes associated with cancer promotion including NOX-2, NF-κB, SP-1, MMP2 and MMP9, B-cell lymphoma-extra large (Bcl-xL); (2) phosphorylation of ERK 1/2, p38 MAPK, STAT-3 and 70-kDa ribosomal protein S6 kinase (p70S6K1); (3) down regulation of NRF-2, Bcl-2 (anti-apoptotic protein), p53 (tumour suppressor) expression; and (4) activation of PI3K/protein kinase B (AKT) pathway (shown in Figures 1 and 2). Almost all of the RAGE downstream factors activated upon AGE-RAGE binding are involved in the development, progression and metastasis of cancers. AGE-RAGE conjunction trigger HIF-1α expression, which enables the cancer cells to acclimatise to surrounding cellular milieu by promoting augmented glycolysis and hence glucose metabolism, EMT and hence events of invasion and metastases, and finally angiogenesis to facilitate all the malignant phenomena [31]. AGE-rich diet fed mice model of prostate cancer displayed RAGE-mediated sustenance and augmentation of migratory capacity of cancer cells. But RAGE negation in tumour stroma hindered the AGE-driven cancer expansion. Hence AGE exposed cancer cells elicited RAGE in tumour-associated stroma thereby triggering cancer development and metastasis. AGE-rich diet incite oncogenic progression via molecular interplay of tumour micro-milieu [39].

Multiple studies have revealed the insinuations of glycation modified HDL, glucose, glyceraldehyde and MG AGEs in facilitating the survival, proliferation, invasion and metastasis of different cancers including that of breast, colorectal, oral, prostate, lung and melanoma [12]. Age-pertinent physical alterations in the ECM regulate cancer cell motility, invasion and metastases. Furthermore, epigenetic changes like chromatin mutations are related to oncogenic gene/protein expression, facilitating metastatic advancement [31]. Concomitance of AGEs with age-pertinent alterations in ECM and inducing epigenetic shift via non-enzymatic covalent modifications (NECM) in histone and chromatin are well studied [60, 61]. Augmented RAGE expression bestow metastatic phenotype to cancer cells [53].

AGE-RAGE signalling prompted interaction between breast cancer cells and cancer-associated fibroblasts (CAFs), via up-regulation of IL-8 (pro-inflammatory cytokine) levels in CAF with ensuing paracrine stimulation of CXCR1/2 (chemokine receptors). This AGE-RAGE driven IL-8/CXCR1/2 instigation in CAFs incited the attainment of characteristics pertaining to migration and invasion in MDA-MB-231 breast cancer cells *in vitro* [62]. Involved in the crosstalk between cancer cells and the concomitant stroma, AGE-RAGE conjunction cues play crucial role in the re-structuring of tumour micro-milieu for metastatic events.

Being at the ultimate cutting-edge phase of cancer treatment, targeting metastasis with highly efficient therapeutic measures is indispensable, for which AGEs and RAGE serve as the most befitting targets of optimization.

## Metabolic evolution and apoptotic evasion via AGE-RAGE synergy

Cancer cells possess distinct ability to rigorously maintain and survive under high oxidative burden, unlike normal cells which undergo apoptotic cell death upon ROS induced DNA damage. AGE-RAGE duo insinuate oxidative stress besides sustained inflammation in oncogenic micro-milieu. In a niche inculcated with metabolic and oxidative stress, RAGE precisely propagates the survival signals in tumour cells by augmenting autophagy/programmed cell survival and attenuating apoptosis/programmed cell death. The synergy existent between AGEs and RAGE aids in the evasion of apoptosis and elicitation of cancer progression, even in high ROS niche by switching to autophagy [59]. Activated glyoxalase GLO-1 enzyme, the de-toxifying defense system serves to sustain survival signals in cancer cells despite abundant glycative and oxidative stress, via AGE-RAGE mediated cues. Evasion of programmed cell death, one of the characteristic hallmarks of cancer cells is facilitated by AGE-RAGE and RAGE-HMGB1 interactions, by switching to the programmed cell survival mode termed autophagy [63, 64]. During chemotherapeutic intervention, the unprecedented rise in autophagy via AGE-guided RAGE activation aids cancer cells in evading death and hence induces therapy resistance. RAGE evokes autophagy concomitant with attenuation of mammalian target of rapamycin (mTOR) phosphorylation and augmentation of Beclin 1-Vps34 (a PI3K) interaction, and impedes apoptosis via p53-transcription independent mechanism. Apoptotic evasion by RAGE is evidently invoked via p53 attenuation, since RAGE depletion augments caspase-3 activity and facilitates apoptosis, while p53 antagonist suppresses caspase-3 stimulation and cell death in the same RAGE depleted cells, ensuing chemotherapy in pancreatic cancer cells *in vitro* [64].

In addressing the metabolic addiction of cancer cells [63] and attributing the metabolic re-programming to cancer treatment [54], two major factors underlie the intricate drug resistant mechanism of cancer cells, namely, (1) evading canonical cell death mechanism, hence evoking cell survival in cytotoxic stress conditions; and (2) cell metabolic re-wiring to fulfil the proliferative needs of cancer cells. AGEs incite RAGE along with HMGB1 promoting oxidative stress and tumour pertinent inflammation. The inflammatory cascade instigated upon RAGE activation is central to the evasion of apoptosis by cancer cells [63], and also crucial to the generation of ATP, hence sustenance of mitochondrial bio-energetics in cancer cells [34]. RAGE prompted IL-6-driven ATP generation and cell proliferation, and also promoted autophagy (LC-3I and LC-3II) in pancreatic cancer cells [65]. HMGB1 invoked by RAGE, plays a crucial role in the regulation of apoptosis and autophagy, arising from metabolic stress [64].

As can be seen, both the crucial factors of apoptotic evasion and metabolic insurgence are pronouncedly promoted by activated RAGE pathway, and thereby RAGE plays a great role in eliciting drug resistance in tumour cells. RAGE intervention is indispensable to address the metabolic addiction and re-programming of cancer cells, and hence overcome drug resistance to conventional cancer treatment [63]. RAGE suppression markedly enhanced sensitivity of cancer cells to cytotoxic drugs, ultra-violet (UV) radiation and hypoxia, with parallel rise in cleaved caspase-3 [64]. Coupling RAGE inhibitors with gemcitabine impeded tumour growth by suppressing autophagy and stimulating apoptosis in pancreatic cancer cells, implanted in the pancreas of C57BL/6 mice [66]. RAGE expression provokes cancer cell survival ensuing metabolic and genotoxic stress events. RAGE and its ligands form a direct connection between inflammatory mediators in the oncogenic micro-milieu and resistance to apoptosis [67].

## Metabolic alterations and epigenetic ramifications of glycation in cancer

Epigenetic adaptations as inheritable alterations but without actual changes in the DNA sequence, are central to maintaining the tissue specific expression of genes. Disruption of normal epigenetic niche by any means disturbs genomic stability and function, with ensuing incitation of malignant transformation. In par with the vital post-translational modifications of histone like arginine methylation, which are central to the maintenance of chromatin dynamics and sustenance of genetic code via transcription processes, MG-induced modifications of arginine and lysine residues of histone proteins are also existent, with deviant implications in cancer by provoking aberrant gene expression [68]. Hence, the glycolytic superfluity of glycated products in cancer form the conduit between altered metabolic flux and aberrant histone modification, thereby offering the link between cancer concomitant shift in cell metabolism and epigenetic cues.

Glycation as a predominant NECM is pertinent to a number of pathological conditions. Histone proteins (rich in lysine and arginine) are especially prone to these glycating alterations, owing to their long half-lives and disarrayed nucleophilic tails. The non-enzymatic post-translational alterations in histone proteins are highly vital to the maintenance of epigenetic landscape, cell fate and genetic code, with implications in human diseases. Glycation forms the most predominant of non-enzymatic alterations in histone. Besides disrupting nuclear protein integrity, histone-chromatin interaction and vitally located amino acid residues, glycation also generates neo-epitopes eliciting immune reaction and impairing normal cell function. Glycation-altered histones being highly immunogenic, generate auto-antibodies to H1, H2A and H2B, reported in different cancers including cancers of head and neck, lung, breast, oesophageal, gastric, gall bladder, colorectal and prostate, indicating the repercussions of histone glycation on its immunogenicity [21]. The glycation of histones culminating with the generation of auto-immune response and genomic instability in cancers cannot be underestimated [12].

The molecular interplay of AGEs by glycation of nuclear proteins link metabolic alterations with epigenetic dysregulation in cancer [48]. It was distinctly recorded with histone glycation mediated perturbations to the nuclear components with localised as well as widespread ramifications in cancer. The detrimental consequences of histone (H3 and H4) glycation by disruption of (1) chromatin compaction; (2) cluster and stability of nucleosomes; and (3) enzymatic post-translational modifications of histone, have been reported. Of the core histone proteins (H2A, H2B, H3 and H4), H3 was identified as the main glycation substrate for reactive agents like MG. Induction of DNA damage, genotoxicity, mutagenicity and immunogenicity by glycation of DNA have already been reported [15, 69, 70]. Furthermore, glycation of nucleosomal DNA besides histone was observed with resultant induction of histone-histone and histone-DNA crosslinks, and perturbations in histone-DNA interactions, thereby inciting powerful damages to chromatin structure. Glycation events by MG also interrupt the histone acetylation processes. Hence, rather than just the sequential impact of metabolic perturbations like AGEs in transforming cancer cell signalling pathways, greater is the direct implication of metabolic alterations on epigenetic micro-milieu via glycation of histone by AGEs (like MG) inciting drastic shift in chromatin architecture and histone post-translational modification signature, thereby affecting chromatin function and gene transcription.

Three-fold increase in histone-AGEs proportionate with increasing age and diabetes have been reported in experimental studies involving diabetic rats. As metabolic memory observed in normal breast tissues with prolonged exposure to dietary AGEs, persistent hyperglycemic memory prevalent in cancer and diabetes incite histone modifications [71, 72]. Although DJ-1 provides the escape from metabolic impact of epigenetic dysregulation in breast cancer cells by protecting histones from glycation, MG was found to modify histone proteins at arginine sites in closer proximity to DNA in breast tumour cells [68]. Mass spectrometric analysis of MCF-7 cells revealed 17 glycated sites, majority in arginine and functional domains [49]. Recent study registered the glycation of more than 100 proteins in hepatic cancer cells, encompassing transcription factors, splicing factors, histones, DNA- and RNA-binding proteins, HSPs and more importantly, enzymes involved in glucose metabolism [17]. DNA and histone crosslinks arising from the glycation modifications via exposure to AGEs alter the electrostatic interactions between the same and also hinder canonical alterations on the corresponding site. These epigenetic instabilities incited by glycation detrimentally influence the enzymatic regulation of metabolic pathways, otherwise normally regulated by DNA and histones, further impairing epigenetic control [20]. Furthermore, AGEs in combination with tumour necrosis factor (TNF) stimulated MMP9 promotor demethylation via “a member of the stress-responsive family of genes called GADD45a” (growth arrest and DNA damage-inducible 45a) [73]. Maksimovic and David [60] emphasise the need for maintaining accuracy of epigenetic information even after many cell divisions, which is otherwise disrupted by the detrimental accretion of glycated adducts on genome-pertinent macro-molecules in the cell. Hence glycation can affect a lot of epigenetic and metabolic processes by modifying vital amino acid residues in metabolic enzymes, histones, DNA and RNA molecules. Glycolytic by-products like glyoxal and MG, tamper with the gene transcription processes, mostly in cells devoid of the detoxifying GLO-1 enzyme [60]. These studies underline the significant impact of metabolic perturbations on epigenetic dysregulation, with the disrupted epigenetic facade in turn affecting metabolic events.

## Metabolic by-products and tumorigenic inflammation

The complex relationship between metabolome and inflammation in cancer was established by Coluccio et al. [26], by assessing the BDCs or liquid biopsy of patients with cancer for the presence of different levels of glycation adducts and cytokines in different stages of the disease compared to the healthy counterparts. They derived a specific pattern of cytokines secreted and glycated adducts generated in different stages of the disease, which were overall higher than the healthy control group [26].

Both MGAs and cytokine panel corresponding to disease stage and tumour proliferation grade were detected in the BDCs secretomes of patients with cancer. To underline, in the BDCs secretome of patients with advanced stage cancer, augmented levels of MGAs along with IL-1β, IL-10 and VEGF were detected, corresponding with the hypoxic, immuno-suppressive and vasculogenic actions respectively, all characteristic of metastatic cancer cells. Hence, the rise in glycolytic by-products like MG upregulate RAGE and further NF-κB, thereby concurrently inciting the cascade of ILs. Rise in the degree of metabolic switch in terms of MG generation surges IL production, proportionate to the increase in disease severity, ranging from local to advanced stage. Increasing with cancer progression, the concentration of MGAs positively correlated with the secretion of ILs: IL-1β, IL-2, IL-4 and IL-6. Lesser concentrations of MGAs and cytokines (ILs) were observed in localized cancer phase. When analyzing the liquid biopsy of cancer-afflicted patients for glycated adducts and secreted cytokines in diverse stages of cancer, the glycation adducts were relatively higher in cancer secretomes than the healthy counterparts [26].

The metabolic shift of tumours attribute to glycolytic superfluity along with an oxidative micro-milieu. This undeniable metabolic dependence of cancers on glycolysis bring forth unintended abundance of glycated adducts, which could be seen in bio-components as micro-molecular as DNA and histones to as macro-molecular as proteins like albumin and collagen. This universal presence of glycation products in the tumour and tumour-pertinent niche call for RAGE activation, which inexplicably restructures the cellular architecture of micro-milieu, favouring migration and metastasis of cancers. AGE-RAGE conjunction downstream incites NF-κB, the major driving factor of inflammation, besides augmenting the expression of DAMP like HMGB1 and S100 group of proteins, by prompting the binding of NF-κB to RAGE promoter and AP-1 to HMGB1/S100 promoters, respectively. RAGE being a strong instigator of NF-κB activation, perpetually generates transcriptionally active NF-κB via *de novo* synthesis of v-rel avian reticuloendotheliosis viral oncogene homolog A (*RelA*) messenger RNA (mRNA). Through NF-κB, AGE-RAGE duo up-regulate the transcriptional expression of genes pertinent to inflammation comprising *E-selectin*, intercellular adhesion molecule-1 (*ICAM-1*) and vascular cellular adhesion molecule-1 (*VCAM-1*), and stimulate production of these adhesion molecules and pro-inflammatory cytokines—IL-6, IL-8 and TNF-α (shown in Figure 2). DAMP molecules are secreted by immune cells under conditions of inflammation, necrosis, injury and hypoxia [12]. These DAMP molecules are pro-inflammatory mediators related to acute and chronic inflammation in cancers.

Besides DAMP molecules, AGE-RAGE binding also induce NOX-2 and ROS production, prompting oxidative stress, further fuelling the inflammatory niche. RAGE acts an oncogene upon ligand binding and activates the inflammatory mediators—Ras and NF-κB, establishing the metabolic paradigm of tumour and adjacent micro-milieu [74]. AGE/RAGE/NOX-2/NF-κB is a self-sustained signalling axis, found upregulated in cancers, upholding inflammation and oncogenic cues [16, 27]. AGE-RAGE driven surge in pro-inflammatory cytokine IL-8 in CAFs, activating CXCR1/2 receptors, incite deregulated inflammatory micro-milieu leading to cancer progression [62].

Inflammation as the central pathway mediating diabetes, obesity and cancer, is by itself fuelled through glycation and oxidative stress processes. AGEs and RAGE as the prime facilitator of the “inflammaging” phenomenon, is known for their pleiotropic pathogenic role in diabetes, obesity and cancers (and many other inflammation pertinent diseases), via its unhindered sustenance by glycation and oxidative stress [75, 76]. RAGE is linked with development of cancers in patients with obesity and diabetes, by dysregulation of insulin/IGF signalling and promotion of pertinent meta-inflammation. RAGE serves as the linking factor between metabolic impairment or metabolic stress (insulin resistance) and inflammation, in obesity and diabetes [77]. Augmented mortality rate in breast cancer-afflicted patients with pre-existent metabolic disorders like obesity and diabetes, via impaired IGF-1 cues acting via S100A7/RAGE pathway, thereby promoting cancer-related angiogenesis in breast cancer micro-milieu, has been reported [78]. Hence RAGE is associated with cancer incidence and increased cancer mortality rate in obese and diabetic patients by influencing dysregulated IGF-1 signalling and sustained inflammation.

Consequently, the generation of AGEs via glycolytic abundance link the metabolic shift of cancers to sustained inflammation via RAGE activation (AGEs-RAGE-NF-κB, AGEs-RAGE-HMGB1/S100, AGEs-RAGE-NOX-2, AGEs-RAGE-IL-8-CXCR1/2 and RAGE-IGF-S100A7) in cancers. The augmented presence of RAGE in the invasive front [79] and AGE levels in the advanced disease stage [26], along with the concurrent rise in different cytokines, confirm the concomitance of AGEs and RAGE with the metastatic and malignant nature of cancers, while simultaneously establishing the connection between metabolic switch of cancers to inflammation via AGEs-RAGE conjunction in cancers. Furthermore, the RAGE-HMGB1 interaction generate inflammatory cues, implicated in significant ATP production via upregulation of mitochondrial bio-energetics [34]. Hence the metabolic shift of cancer cells via AGEs can be attributed to instigation of inflammation via RAGE and HMGB1, which in turn fuel the metabolic activity of cancer cells for accelerated energy generation. Stimulation of RAGE signalling axis subsidises the concomitance of metabolic syndrome like obesity, hypertension, hyperglycemia and diabetes with cancer growth by driving inflammation and oxidative stress, ideal for oncogenic events in the micro-milieu [80].

## AGEs-RAGE and tumour micro-environment: immune evasion

AGE-RAGE duo insinuate oxidative stress besides sustained inflammation in oncogenic micro-milieu. NADPH pathway is involved in oxidative stress concomitant RAGE activation [81]. Extracellular AGEs bind RAGE and facilitate downstream augmentation of NOX-2, ROS production and NF-κB. The ROS enriched tumour micro-milieu favours the oxidation of HMGB1, besides other proteins, lipids and DNA, thereby facilitating the undesirable effects of this protein on immune suppression and cancer progression. Besides directly activating transcriptional expression of HMGB1 via RAGE, AGEs also incite HMGB1 oxidation by nurturing an oxidative stress-rich micro-milieu. Hence the metabolic impairment of cancer cells in addition to generating AGEs, culminate in the arousal of molecular events conferring immune tolerance.

High amounts of HMGB1 cytoplasmic (mRNA) expression (with ensuing cellular release) were detected more often in basal-like or triple-negative breast cancers, than the hormonal and HER2 counterparts, with considerable pertinence to adverse consequences in patients with cancer [82]. This tumor-specific cytoplasmic expression of HMGB1 confers immune tolerance and poor prognostic value, by serving as a tolerogenic signal in RAGE—reliant manner in extracellular oxidised form. Hubert et al. [82], reported pro-tumour/immuno-suppressive effect of HMGB1, and suppression of tumour development ensuing stimulation of adaptive immune responses via profound re-modification of immune micro-milieu by HMGB1 inhibition, thereby enhancing the efficacy of [anti-programmed cell death ligand 1 (PD-L1)] mono-immunotherapeutics in cancer.

In many tumours, stromal cells incite CAFs and vascular endothelial cells, and employ multiple supportive immune cells like TAMs and MDSCs, ultimately creating an immuno-suppressive and inflammatory tumour micro-milieu. The crosstalk between cancer cells and immune cells drive tumour progression through RAGE-mediated active expression of inflammatory cytokines—IL-6, IL-1β, TNF-α, other cytokines and chemokines, which elicit the secretion of pro-inflammatory RAGE ligands, comprising HMGB1 and S100 family of proteins [83]. HMGB1 released from immune cells in a persistent inflammatory milieu instigate chemokines and cytokines, which mediate the CAFs, MDSCs and TAMs to create inflammatory and immuno-suppressive onco-milieu by preventing the entry or T-cells and natural killer (NK) cells, conducive for the evasion of immune detection, thereby facilitating unchecked tumour growth. This interplay of AGEs-RAGE via HMGB1 and other pro-inflammatory molecules in TME forms the basis of immune evasion and cancer survival, further leading to ECM alterations via AGE-RAGE crosstalk in TME paving way for invasive phenotype and metastatic dissemination (as shown in Figure 2).

## AGEs-RAGE and TME: glycation, inflammation and metastasis

The TME is formed by multiple cell types held together in a modified ECM. The ECM, not just serve as a tumour cell support, but also as a mediator of cell-cell and cell-matrix interactions. ECM is modified by ROS and proteases released from the cancer and supporting cells, under the influence of pro-inflammatory ligands and molecules, incited by tumour-pertinent conditions of acidosis, hypoxia, injury, inflammation and immune response, all either stimulated by or stimulating glycolytic abundance (Warburg’s metabolism), coupled with AGEs as well as lactate over-production. Cancer-pertinent ECM modifications are concomitant with poor diagnosis, and the potentially modified ECM protein signatures could act as prognostic biomarkers [84]. Considering the significance of ECM proteome in cancer, AGE-mediated ECM alterations cannot be undermined. Accretion of AGEs can have strong implications on TME in invasion and metastatic dissemination of cancers via potent RAGE-reliant molecular cues and ECM alterations, and RAGE-independent ECM changes brought on directly by glycation and oxidative stress. Furthermore, AGEs and AGE-activated RAGE, linking glycation and inflammation, could potentially serve as metabolic and molecular signatures of malignant or metastatic cancers, and hence can be employed as prospective biomarkers, pertinent to cancer stage and individual tissue-specific expression.

AGEs and RAGE function together as instigators of molecular and cellular interplay between cancer cells and the associated stromal component. This interplay between cancer and stromal cells is highly crucial with respect to the elicitation of malignant features in cancer cells. Generally, tumours maintain the niche conducive for their growth by sustaining an inflammatory micro-environment. This sustenance of inflammatory tumour micro-milieu is facilitated by AGE-RAGE and HMGB1/S100-RAGE signalling. Existence of AGEs in the tumour milieu initiates a signalling cascade which activates RAGE and augments the expression of DAMP molecules/pro-inflammatory ligands—HMGB1 and S100 family of proteins, by prompting the binding of AP-1 to HMGB1/S100 promoters. AGEs further invoke the binding of NF-κB to RAGE promoter, and HMGB1/S100 provoke the binding of SP-1 to RAGE promoter, thereby synergistically augmenting RAGE mRNA and protein expression. Extracellular AGEs, besides prompting AGEs-RAGE-HMGB1-S100-NF-κB cascade, also incite AGEs-RAGE-NOX-2-ROS-NF-κB molecular cues, eventually linking the AGEs-RAGE pathway to sustenance of inflammatory milieu by multiple facets (NOX-2; ROS) [12].

Inflammatory events set in motion via RAGE axis instigate carcinogenesis. Yet again the sustenance of this inflammatory milieu by AGEs-RAGE axis in developing cancers, marks the niche for malignant transformation. By paracrine signals, tumour cells stimulate the stromal cells to secrete pro-inflammatory ligands and molecules, which aid in their acquisition of malignant characteristics, starting with EMT. AGE-RAGE molecular cues incite a dysfunctional interplay between cancer cells and the pertinent stroma towards attaining malignant characteristics. AGEs via RAGE trigger ROS generation, along with ERK and AKT phosphorylation in CAFs. IL-8 is the pro-inflammatory chemokine, highly augmented in CAFs of tumour milieu, upon AGEs-RAGE activation, by c-Fos dependent regulation. This IL-8 from CAFs via paracrine signals prompt CXCR1/2, in turn resulting in the derivation of migratory and invasive characteristics in cancer cells [62]. Hence the AGEs-RAGE signalling in CAFs, one of the most vital components of TME, plays crucial interplay between the CAFs and cancer cells by instigating AGEs-RAGE-ROS-ERK 1/2-AKT-c-Fos signal conversion cascade, resulting in the expression and release of IL-8, which by paracrine signal driven chemokine receptor (CXCR1/2) engagement prompts malignant features in cancer cells.

Exposure to dietary AGEs can stimulate oncogenic transformation in otherwise normal cells. However, the exposure of already formed cancer cells to dietary AGEs can bring forth even more detrimental effects like highly augmented cell proliferation, moderate oxidative imbalance, inflammation, pro-survival signals, and ultimately extensive ECM remodelling, paving way for metastasis. Hence AGEs, whether endogenous or exogenous, have inherent potential to trigger oncogenesis in otherwise normal cellular milieu, and evoke metastasis in pre-existing tumour milieu. AGEs activate RAGE-NF-κB and AKT-mTOR signalling pathways in cancer cells, forming the strong link between dietary AGEs and aggressiveness of colorectal cancer, by inciting inflammation and metastasis [85]. In cancer cells, AGEs elicit (1) phosphorylation of AKT and mTOR (a serine/threonine protein kinase in the PI3K-related kinase family), responsible for cancer cell proliferation and pro-survival signals; (2) phosphorylation of GSK-3β, responsible for ECM remodelling and hence metastasis; (3) expression of MMP1, 3, 7, 9 and 10; activation of MMP2 and 9, mediators of ECM and metastasis; (4) rise in cytokines IL-1β and IL-8, responsible for inflammation; (5) augmentation of RAGE and NF-κB p65, and IκB suppression, responsible for inflammation and metastasis.

The glycolytic switch of cancer cells (aerobic glycolysis—Warburg effect) mounting up the production of glycation products (AGEs) like MG is responsible for the metastatic or malignant switch of cancer cells. MG AGEs have been proved to augment the invasiveness of (breast, colon, prostate, pancreatic and glioblastoma) cancer cells, thereby bestowing them with malignant features and making them biologically aggressive as shown in Table 2 [25, 26, 86, 87]. MG besides eliciting invasive effects in cancer cells, incite alterations in ECM, and enhance TGF-β, brevican, CD44 and tenascin C (glycoproteins), the components of ECM pertinent to invasion process. Hence glycation alters the expression of ECM components (cell-cell adhesion molecules and matrix-degrading enzymes) to suit the invasive process of cancer cells. This emphasises the role of AGEs in the crosstalk between ECM components and cancer cells to promote metastasis. Pan et al. [45], established the concomitance of AGEs and breast cancer metastasis via instigation of RAGE/Toll-like receptor 4 (TLR4)/myeloid differentiation primary response factor 88 (MyD88) signalling with up-regulated MMP9 expression and nuclear translocation of NF-κB, reduced IκB expression [45]. Hence glycation/AGEs are directly linked with metastasis of cancers. It is to be noted that earlier studies identified the expression of argpyrimidine in breast cancer fibroblasts, and carboxymethyl lysine in colon cancer-pertinent tissues of patients with cancer [88]. Nevertheless, many studies involving both diagnostic and therapeutic application of AGEs in cancers, as shown in Tables 2 and 3, focus on the carboxymethyl lysine AGEs, even the most recent works [87], and very few studies have analyzed the interplay of argpyrimidine AGEs in onco-theranostic implications. Hence we underline the need for further studies researching the expression and impact of argpyrimidine in cancer cells as well as the tumour-pertinent stroma cells forming the TME, to better understand the combined role of prominent AGEs in eliciting cancer metastasis and develop combinatorial therapeutics targeting the same.

Rojas et al. [89] have lately explained the dual role of RAGE in the elicitation and sustenance of inflammatory milieu, with not just in the development and dissemination of cancer cells, but also in the inflammatory reaction to *Helicobacter pylori* (*H. pylori*) infection by acting as a PRR. Hence cancer cells via AGEs and RAGE prepare the tumour micro-milieu (including CAFs and ECM mainly) for metastasis, by maintaining (Warburg’s metabolism) aerobic glycolysis-mediated upsurge in glycation and subsequent inflammation processes, besides preparing the cells to meet surplus energy demands with the metabolic reprogramming. Therefore, as we emphasise, the metabolic switch of cancer cells plays crucial role in the metastatic shift of cancer cells. Furthermore, the interplay of AGEs-RAGE axis in elicitation of cancers involving micro-organisms also needs to be studied, considering the pertinence of RAGE as PRR in *H. pylori* infection-related gastric and gut cancers.

## Glycation/AGEs and gut microbiome in cancer

**Figure 3:**
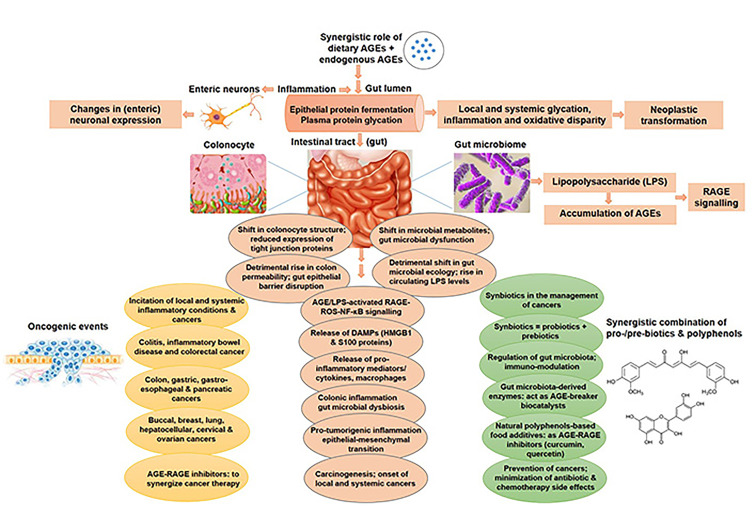
AGEs-mediated oncogenic alterations in intestinal micro-milieu and gut microbiome. Accretion of AGEs, both dietary/exogenous and endogenous AGEs, incite multiple cellular and molecular events, including protein fermentation, glycation, inflammation, colonic permeability, gut barrier dysfunction, pro-inflammatory cytokines/ligands by AGEs-RAGE-ROS-NF-κB signalling, augmented LPS circulation levels, gut microbial dysbiosis, all leading to the onset of glycation- and inflammation-induced cancers, both local and systemic, and ultimately malignant transformation. Synbiotics and natural polyphenols can be used in combination to curb the side effects of conventional chemotherapy, and those with AGE/RAGE inhibitory potential can be exploited to synergize cancer treatment. LPS: lipopolysaccharide

RAGE belongs to the family of PRRs, encoded in the MHC class III region of MHC. Burgueño and Abreu [90] emphasise the immune response and inflammation elicited by dysregulated expression of TLRs and PRRs like RAGE in intestinal epithelium, resulting in onset of gut dysbiosis, eventually augmenting predisposition to colitis and tumorigenesis. The receptor RAGE once stimulated by AGEs or other ligands, ultimately elicits inflammatory response via NF-κB. This paves the way for impaired immune function and sustained inflammation, thereby predisposing to potential infections, inflammatory conditions like Crohn’s and ulcerative colitis, and even colitis-related colorectal cancer [90]. Exposure to dietary AGEs by themselves can inflict damages to the gut microbiome, possibly creating “metabolic memory” by generating glycation adducts in long-lived tissue proteins and producing an imbalance in the cellular homeostasis or inducing dysbiosis. These AGEs also wreak havoc by binding and activating RAGE receptors in epithelial cells lining the gut, thereby promoting tumorigenic inflammation via pro-inflammatory cytokines (shown in Figure 3).

Maillard or glycation reactions occurring in the intestine disrupt the homeostasis of gut microbiome and damage the gut bacterial species, by accruing glycation adducts/protein aggregates in the bacteria. Dietary AGE exposure impair the components of regular gut microbiota, boosting non-enzymatic glycation in plasma and systemic pro-tumorigenic inflammation [91]. Reddy et al. [91], revealed that intervention with AGE inhibitors can enhance the gut bacterial lifespan, thereby preventing the dysbiosis and inflammation arising from AGEs, ultimately nullifying the impending propensity for colorectal cancers.

Interaction between SjE16.7 (calcium binding protein) secreted from the eggs of *Schistosoma japonicum* and RAGE in host cells, with resultant instigation of NF-κB signalling pathway, ROS generation and pro-inflammatory cytokine (IL-6 and TNF-α) secretion, stimulated colorectal cancer development in mouse model. SjE16.7 is a neutrophil-attracting and MAC-stimulating protein, with role in inflammatory reaction in schistosomiasis. Hence not just the disruption of gut microbiome, but also parasitic incursions like schistosome infection (a helminth antigen) lead to the onset of cancer via RAGE activation in the human host [92].

As one of the most recent causes for concern in cancer progression, it is indispensable in maintaining not just biochemical parameters but also the normal gut microbiome for preventing unnecessary propensity to oncogenic events by infection or inflammation arising from the imbalance in gut microbiota.

Furthermore, bacteria have been reported in tumours constituting the tumour microbiome. Tumoral bacteria are metabolically active, exerting changes in the chemical structure of certain chemotherapeutic drugs and disturbing treatment response. Specifically anaerobic bacteria thrive in hypoxic and others in vascular-rich tumour micro-milieu. Resistance to gemcitabine (a chemotherapeutic drug for gastro-intestinal cancers) is elicited by gamma-proteo bacteria present in pancreatic cancers. Chemo-resistance in colorectal cancer cells is facilitated by *Fusobacterium nucleatum* via instigation of autophagy and stimulation of TLRs present on cancer cells. Intratumoral bacteria control immune reactions, a few elicit anti-tumoral immunity, while most others evoke immuno-suppression, directly altering the immunotherapy response [31].

Aschner and others have emphasised the substantial impact of dietary/exogenous AGEs on the diversity and richness of gut microbiota, whose features are strongly concomitant with accretion of AGEs in the host [93]. Bacterial endotoxin—LPS (cell wall component of gram-negative bacteria), a potent pro-inflammatory agent regulates the interplay between gut microbiome and detrimental effects of AGEs, by instigating systemic inflammation via activated RAGE-ROS-NF-κB signalling (shown in Figure 3). Dietary AGE-driven modifications in gut microbiota incite disruption of intestinal epithelial barrier, augment plasma and tissue AGEs, with ensuing rise in circulating levels of LPS. Chronic exposure to LPS augment accretion of AGEs. Hence AGEs and LPS stimulate each other in a positive feedback loop to impair gut microbiota and sustain inflammation, by releasing pro-inflammatory cytokines, chemokines, mediators, adhesion molecules and ligands (HMGB1 and S100 proteins) in the micro-milieu. Recent studies have revealed the alteration in composition of gut microbiota and induction of insulin resistance, along with subsequent shift in colonocyte structure, rise in colon permeability, reduction in expression of tight junction proteins, ultimately leading to intestinal epithelial barrier dysfunction, with resultant augmentation of RAGE NF-κB signalling upon exposure to dietary AGEs [94–96]. The most interesting fact recently put forth by Reddy et al. [91] is that Maillard reactions are detected in the gut bacterial species also besides the host, generating pathogenic protein aggregates in the bacterial species [91].

AGEs from diet modify the gut microbiota with elicitation of both local and systemic (colonic/gut inflammation) pro-inflammatory signalling and endothelial dysfunction, further leading to pro-tumorigenic inflammation and potential incitation of different cancers, both local and systemic, including buccal, breast, lung, hepato-cellular, gastric, gastro-oesophageal, colon, colorectal, pancreatic, ovarian and cervical cancers [97]. AGEs markedly reshape the gut microbiota profile, inducing gut microbial dysbiosis and exerting detrimental impact on colonic epithelium and enteric neurons, by augmenting protein fermentation/glycation and attenuating neuronal expression, respectively, paving way for inflammatory conditions like colitis, inflammatory bowel disease and even colo-rectal cancer [96]. Evidence for reversibility of dietary AGEs-induced rise in plasma and tissue AGEs, along with the elimination of colonic inflammation and regaining gut microbial composition, have been provided by van Dongen et al. [98] with switch to low-dietary AGEs. Considering the fact that plasma and dietary AGEs have been associated with breast and colorectal cancer risk (as shown in Table 2), we suggest the pre-meditated screening for plasma AGEs or skin AGE fluorescence in high-risk population, with measures for reversal of pro-inflammatory clinical presentations in those detected with high AGE levels, by switching to a diet low in AGEs.

AGE-impacted gut microbes detrimentally affect the cell signalling (AGE-RAGE-ROS-NF-κB), cell permeability (reduced tight junction proteins), immune system (secretion of pro-inflammatory cytokines-IL-6/8, MACs, immune cells, etc.) and metabolic patterns (insulin resistance). All these cellular events contribute to inflammation and instigation of EMT to ultimately bring forth carcinogenesis [97]. Thekkekkara et al. [97], suggest that the pro-biotics and anti-oxidants can be utilised to synergistically treat cancers, and also restore the damaged intestinal barrier, incite immuno-modulation and prevent cancer. Hence, we advocate the synergistic combination of synbiotics (pro-and pre-biotics) and plant-based polyphenols with anti-glycation activity (natural AGE inhibitors) to address the synergistic role of exogenous (dietary) and endogenous AGEs in prompting the inflammatory cascade pertinent to cancers. Furthermore, we emphasise that this synergistic combination of synbiotics and anti-glycative polyphenols can aid in attenuation of side effects pertinent to chemotherapy, radiotherapy, surgery, and antibiotics administered post-surgery, possibly by targeting AGE-RAGE signalling cascade, since RAGE inhibitors are already tested in phase I/II clinical trials to suppress the adverse effects of chemotherapy (shown in Table 3).

## Implications of anti-glycation agents/AGE inhibitors in cancer

Augmentation of AGEs plays a crucial role in the pathogenesis of ageing and age-related diseases such as diabetes, cataract, Alzheimer’s, Parkinson’s, ALS, atherosclerosis, stroke, cardiovascular disorders, chronic obstructive pulmonary disease (COPD), arthritis, retinopathy, neuropathy, nephropathy (renal failure), cancer and pre-mature ageing [99–101]. Pathogenic signalling of AGEs occur via RAGE-receptor dependent as well as receptor independent mechanisms. AGE-RAGE pathway has significant implications in inflammation, degeneration, diabetes and ageing [75, 102, 103]. AGEs via molecular interplay with RAGE ignite a cascade of signalling molecules eliciting cytokines and ROS production, causing glycoxidative injuries to nuclear/cellular proteins and DNA, indicative of the receptor mediated pathogenicity of AGEs. Besides RAGE mediated signal execution, AGEs also induce structural and functional perturbations, misfolding and crosslinks of proteins, leading to amyloid-like aggregation and biogenic toxicity [29].

Protein homeostasis is crucial for enhancing longevity, and is affected by protein misfolding causing accretion of insoluble protein aggregates. Hence, glycation alters protein dynamics, causing aggregates and provoking pathological ageing plus degenerative diseases [6]. AGEs besides causing glycoxidative stress mediated damage, also impair the cellular repair systems like ubiquitin-proteasome system (UPS), which dispose the unfolded and oxidised proteins, by blockade of its proteosomal core entry with AGEs cross-linked protein aggregates. In the annual scientific meeting of British Society for Research on Ageing (BSRA) 2012, Prof. Lithgow emphasised that mitigants of protein aggregation such as fibril-binding flavonoid ThT and curcumin could extend the lifespan and alleviate the concomitant pathogenic processes [104]. Alavez and colleagues [105] have displayed efficient pharmacological intervention of age-related changes with amyloid aggregate-binding molecules, resulting in extension of lifespan. Thus, molecules with potential to inhibit protein glycation/formation of AGEs and hence protein aggregates, can potentially interfere with the pathogenesis of ageing and cancer. Ageing-concomitant re-modelling in the structure and functions of brain meningeal membrane via glycation prompted by accretion of AGEs in long-lived protein like collagen present in the meninges (ECM) has been registered recently through recent *in vitro* studies [33].

The multiple glycated adducts as common as MG are widely variant depending on the niche and cannot be sufficiently individually targeted with specific antibodies owing to the variations in the glycating sugar source and glycated protein component. Hence the anti-glycation agents come into play in places requiring generalized and multi-stage inhibition of multiple AGE adducts, with their mechanism of action varying from scavenging free radicals and eliminating oxidative stress to competitively binding with proteins like albumin and protecting them from glycation. The use of dietary polyphenols has been advocated for the regulation of AGE-RAGE axis in the prevention and treatment of cancer (Table 3), by AGE inhibition, blockade of AGE-RAGE ligation, RAGE suppression, mitigation of inflammation and regulation of gut microbiome [106]. Multiple hydroxyl groups of polyphenols confer them the potential to scavenge ROS, trap di-carbonyl species responsible for adduct formation and thereby hinder AGEs formation.

Just like anti-oxidant agents, anti-glycation agents can also have possible paradoxical role in cancer, by supporting as well as suppressing cancer. The antagonistic or protagonistic role of glycation inhibition as a treatment strategy in cancer can clearly be sorted out by observing the impact of AGE inhibitors/anti-glycation agents in cancer cells studied so far [12]. Strong histone glycation and augmented expression of oncogenic protein DJ-1 (an important histone deglycase) in breast cancer cells, calls for the analysis of evasion of ‘glycation-driven death’ by cancer cells via overexpression of DJ-1 [48]. Cancer cells protect themselves from metabolism concomitant cell stress by over-expressing DJ-1, which dynamically deglycates histones. However, unlike the deglycating enzyme, anti-glycating agents do not serve to deglycate the already glycated adducts but rather prevent further formation of glycated adducts and potentially suppress the glycated adducts driven RAGE activation and ROS generation.

Song et al. [107] and Dariya and Nagaraju [108] emphasise the role of natural compounds/phytochemicals like alkaloids, flavonoids, polyphenols, polysaccharides and terpenoids as AGE inhibitors or anti-glycation agents, with potential candidacy for novel cancer drug discovery [107, 108]. We demonstrated that diosmin, a flavonoid exhibiting anti-glycation property (inhibition of argpyrimidine, pentosidine and total AGEs), elicited synergistic anti-tumour activity in TNBC cells *in vitro* in combination with cytotoxic compound plumbagin, a naphthoquinone [109]. Furthermore, we have previously discussed about different phyto-compounds/natural polyphenols like curcumin, quercetin, genistein, glycyrrhizin, resveratrol, epigallocatechin gallate and garcinol, with anti-oxidant, anti-glycation and/or anti-inflammatory properties, simultaneously eliciting cancer cell death, potentially via inhibition of RAGE and/or its ligands [12]. Recently, Jia and other researchers have revealed the mechanistic inhibition of Maillard reaction or formation of AGEs by polyphenolic antioxidants, which are safe and effective with nil to negligible toxicity in healthy cells. They recommend that nutraceutical polyphenols can regulate RAGE expression, suppress MAPK and TGF-β pathway, and successfully promote Kelch-like ECH-associated protein 1 (KEAP1)-NRF-2 pathway [110]. Hence, we highly advocate the exploitation of natural anti-glycation agents, especially polyphenols, for the attenuation of AGE-RAGE pathway, and suppression of this glycation duo in cancer cells/inflammatory milieu, with potential implications as food additives and/or combination drugs with conventional chemotherapy for efficacy enhancement and elimination of adverse effects via dose reduction.

## Diagnostic and therapeutic significance of targeting AGEs and RAGE in cancer

The alarming rise in cancer incidence even among the younger population summons the need for invention of new, enhanced cancer detection and treatment strategies that exploit the innate predominant characteristics of cancer cells distinguishing them from normal cells. One such crucial peculiarity of cancer cells is the rise in glycolytic flux leading to aberrantly surplus accretion of highly reactive sugar metabolites—AGEs (electrophilic species), capable of covalently binding macro-molecules like proteins, with ensuing structural and functional perturbations. Hence, exploiting AGEs or glycated adducts or glycation-modified proteins for cancer diagnosis and treatment (seen in Table 3), could offer exceptional advantage owing to their differential expression in cancer cells. For example, histone H3 glycation adducts were reportedly high in tumour samples compared to corresponding non-tumour specimen from the same patients [48]. Recently, Knörlein et al. [111] recommend exploiting histone glycation in the diagnosis and treatment of cancers. Existence of antibodies to glycated histone with potential immunological insinuations in diabetes and cancer underline the diagnostic and therapeutic relevance of histone glycation in cancer [21].

High expression and activity of RAGE and its ligands strongly impact the invasiveness of cancers, with studies reporting the pronounced positivity of RAGE in the invasive front, thereby augmenting the spread of cancers and attenuating the survival of patients. Palati and colleagues [79] recently reported higher RAGE levels in well-differentiated oral squamous cell carcinoma by assessing many studies covering more than 800 cases. RAGE exhibits potent interplay with HMGB1, augmenting cell motility and thereby promoting tumour invasiveness. Besides RAGE-HMGB1 interplay, AGE-RAGE conjunction also instigate cell migration in cancer cells, with direct repercussions in angiogenesis and metastasis of multiple cancers [24, 112, 113]. AGE-RAGE expression has been directly correlated with cancer malignancy, with higher propensity for metastasis and poor survival in cancer-afflicted patients with diabetes, potentially due to the underlying role of pre-existing AGEs in promotion of cell migration, ERK phosphorylation, RAGE, MMP2 and 9 expression, which were indeed curbed by the use of RAGE antibodies and RAGE interfering RNA (RNAi). RAGE antisense or RNAi and RAGE antibodies have proved to interfere with cell motility, and hence can be exploited for targeted therapy of high-RAGE expressing cancer cells [12]. Hence AGEs and RAGE have potential prognostic implications especially in diabetic patients, and metastatic events can be targeted beforehand with calculated risk assessment and negation of AGE-RAGE levels via molecular interference (as seen in Table 3). Besides AGEs and HMGB1, S100 group of proteins like S100A4 and S100A7 are also implicated in cancer growth, migration and angiogenesis via potent interaction with RAGE and potential re-modelling of tumour micro-milieu [56, 78].

As we already mentioned, AGEs and AGE-activated RAGE, concomitant with glycation and inflammation, could potentially function as metabolic and molecular signatures of malignant or metastatic cancers, and hence can be exploited as potent biomarkers, pertinent to cancer stage and individual tissue-specific expression. To be precise, the administration of AGE-RAGE inhibitors in combination with chemotherapy can be decided based on the individual patient profile identifying the pathological stage of cancer and immuno-histochemical/biochemical studies identifying the person-specific expression of AGEs, RAGE and sRAGE. Hence the AGE-RAGE combinatorial and targeted therapeutics could offer an upper hand in the precise and personalized treatment of cancers. As can be seen in Table 3 and clinical trial data explained later, AGE-RAGE inhibitors are strongly reported to increase sRAGE levels in study subjects, and still more studied in combination with conventional chemotherapy to curb the adverse effects of cytotoxic chemotherapy already in clinical practice, including cardio-toxicity and cognitive decline, and also to achieve efficacy enhancement and dose reduction of the existing chemotherapeutic drugs.

**Table 3:** RAGE targeted diagnostic and therapeutic interventions studied in cancer

RAGE targeted intervention/treatment	Condition/disease	Tumour pertinent effects studied	References
**Nanoparticles**			
DADS-RAGE-SLN: RAGE-antibody conjugated, DADS laden SLN	Breast cancer	DADS-RAGE-SLN elicited substantial rise in pro-apoptotic proteins and parallel reduction in anti-apoptotic proteins with enhanced anti-tumour activity and selective toxicity in MDA-MB-231 breast cancer cells	[114]
Novel nano-therapeutics targeting RAGE	Breast cancer	Target specificity and potent cytotoxicity in TNBC	[115]
CML-HSA conjugated, RAGE targeted multi-modal nanoparticles (^64^Cu-Cy5-G4-CML)	Prostate cancer	Sufficient RAGE targeting established with ^64^Cu-Cy5-G4-CML in LNCaP and DU145 prostate cancer cells, mice xenografts and human samples, confirming the feasibility of RAGE-targeted cancer imaging with AGE-conjugated nanoparticles	[116]
**Nucleic acid-based drugs**			
An aptamer-based RAGE antagonist	Colo-rectal cancer	Decreased cancer cell proliferation, migration and angiogenesis via inhibition of S100B-dependent and -independent stimulation of RAGE/NF-κB/VEGF-A signalling in HCT116 colorectal cancer cells	[48]
Genetic ablation of RAGE—by shRNA and *CRISPR/Cas9 techniques	Glioma	Suppression of growth, invasion and immune evasion of cancer cells by abrogation of galectin-3 and MMP9 expression, and AKT and ERK 1/2 activities in murine glioma model	[117]
RAGE-specific siRNA transfection	Pancreatic cancer	Elicited apoptotic cell death and gemcitabine-mediated cytotoxicity via suppression of PI3K/AKT/mTOR cues in both resistant and non-resistant MIA PaCa-2 pancreatic cancer cells	[118]
**Nanomedicines/antibody-based drugs**			
A monoclonal antibody (mAb; IgG 2A11)—RAGE inhibitor	Pancreatic cancer	Mitigated autophagy and enhanced the cytotoxic efficacy of gemcitabine in murine pancreatic tumours	[119]
RAGE-targeted ADC	Endometrial cancer (EC)	RAGE over-expression in EC adversely correlated with patient survival; RAGE-ADC: 100-fold more effective in EC cells than non-malignant cells; 200-fold more cytotoxic than the drug alone; non-toxic to normal mouse model and substantial tumour growth suppression in mouse xenograft model of EC	[120]
RAGE-targeted ADC	Ovarian and prostate cancer	Demonstrated the efficacy of RAGE-ADC against high RAGE-expressing ovarian and prostate cancer cells *in vitro* and *in vivo* mice models	[121]
**RAGE antagonist**			
FPS-ZM1, a high-affinity RAGE-specific blocker	Breast cancer	Established the efficacy of FPS-ZM1 in blocking the AGEs-induced interaction between CAFs and breast cancer cells, by inhibiting RAGE; AGE-driven IL-8 rise in CAFs, promoting elicitation of invasive/malignant features in cancer cells, was inhibited via blockade of RAGE-ROS-ERK 1/2-AKT-c-Fos signal cascade	[62]
**Pharmacophore**			
3-Styrylchromone derivative: 7-methoxy-3-hydroxy-styrylchromone (a papaverine-mimetic and a novel α-glucosidase inhibitor)	Colon cancer	Anti-inflammatory, anti-proliferative and anti-cancer effects in colon cancer cells via suppression of HMGB1-RAGE-ERK 1/2 signalling Augmented pro-apoptotic Bax and caspase-3/7 expression in HCT116 cells Individual and synergistic augmentation of the above mentioned effects with DNA damaging agents in cancer cells	[83]
**Phyto-compounds**			
Curcumin, quercetin, withaferin A—natural phytochemicals with anti-glycation activity	Many cancers including breast, colon and prostate	Inhibition of various molecular signalling pathways involved in RAGE-driven cues; potentiality in the prevention of diabetes-induced cancers	[122]
Scutellarein—a flavone from the plant *Chrysanthemum indicum*	Colon cancer	Reduced cell viability and enhanced apoptosis via rise in cdc4 and fall in RAGE protein expression, coupled with an upsurge in RAGE ubiquitination in colon cancer cells	[123]
Quercetin—a flavonoid and AGE inhibitor	Pancreatic cancer	Induced cell cycle arrest, apoptosis and gemcitabine sensitivity via attenuation of RAGE expression in resistant MIA PaCa-2 pancreatic cancer cells	[118]
Papaverine—an opiate alkaloid	Fibrosarcoma	Elicited considerable suppression of RAGE-reliant cell proliferation, invasion and migration, RAGE-reliant NF-κB activation and RAGE expression in HT1080 human fibrosarcoma cells Identified as a potent RAGE inhibitor by optimized-peptide strategy of transformation to small molecules	[124]
**Clinical trials involving AGEs-RAGE axis (with implications in cancer)**			
Azeliragon—oral RAGE inhibitor, in combination with dexamethasone, a corticosteroid (phase I trial), in patients with malignant glioma	Glioma and glioblastoma	To mitigate inflammation, thereby reducing the side effects of chemotherapy To inhibit RAGE pathway, thereby reducing cerebral edema post-surgery in patients with glioblastoma To decrease the dose of dexamethasone required by concurrent administration with azeliragon	[125]
Azeliragon or TTP488, formulated as a 5 mg hard gelatin capsule (phase I and II trials), in patients with refractory pancreatic cancer	Metastatic pancreatic cancer	To assess the safety and efficacy of azeliragon in patients resistant to first-line treatment of metastatic pancreatic cancer	[126]
Azeliragon or TTP488 (phase I and II trials), in women with early breast cancer	Non-metastatic breast cancer and cancer-related cognitive decline	To evaluate the impact of azeliragon in attenuation of cardiac toxicity from chemotherapy in women with early breast cancer To target RAGE pathway for subsequent attenuation of anthracycline-associated cardiotoxicity and chemotherapy-associated cognitive decline To assess the safety of azeliragon when given along with chemotherapy	[127]
CX-01 in combination with azacitidine (phase I trial), in patients with MDS and AML	MDS and AML	To treat relapsed or resistant MDS and AML To enhance the cytotoxic effects of azacitidine on MDS and AML hematopoietic stem cells by impeding the interaction of HMGB1-RAGE and HMGB1-TLR4, and chemokine CXCR4 axis	[128]
Grape seed extract (oligomeric procyanidin complex) in combination with vitamin D (phase I trial), in patients with solid cancers	Solid cancers (gastrointestinal, lung, breast, prostate, lymphoma and cancer of the lymph nodes)	To identify plant-based compounds for safe reduction of systemic inflammation in patients with advanced cancer To curb various inflammatory markers including AGEs and sRAGE in the patients	[129]
Vitamin D—50,000 IU D3 (phase IV trial), in women with PCOS	Polycystic ovarian syndrome; vitamin D deficiency	To evaluate the beneficial clinical impact of vitamin D treatment on PCOS with impaired metabolic concomitance Vitamin D augmented serum sRAGE levels in PCOS afflicted women	[130]
DPP-IV inhibitors (sitagliptin) in patients with type 2 diabetes mellitus	Diabetes mellitus	To evaluate the impact of DPP-IV inhibitors on cancer frequency and the underlying AGE-RAGE cues in Japanese patients with diabetes Might work as a cancer protective agent in diabetes by blocking the AGE-RAGE axis	[131]
Relationship between dietary AGEs, inflammation and oxidative stress in breast cancer patients	Breast cancer	To compare dietary AGE intake and serum AGE levels in healthy individuals and in patients with breast cancer To assess the levels of serum carboxymethyl lysine (AGEs), RAGE and sRAGE in the patients	[132]
A low AGE dietary intervention in breast cancer survivors	Breast cancer	To assess the effect of a low AGE diet on weight (BMI), known (IL-6 and CRP) and novel (AGE and RAGE) prognostic biomarkers, and hence post-cancer prognosis	[133]

DADS: diallyl-disulphide; SLN: solid-lipid nanoparticle; Cy5: Cyanine5 dye; shRNA: short hairpin RNA; CRISPR: clustered regularly interspaced short palindromic repeats; siRNA: small RNAi; ADC: antibody-drug conjugate; Bax: Bcl-2 associated X protein; MDS: myelo-dysplastic syndrome; AML: acute myeloid leukemia; PCOS: polycystic ovary syndrome; DPP-IV: dipeptidyl peptidase 4; BMI: body mass index; CRP: c-reactive protein; *CRISPR/Cas9: a unique genome editing technology, which aids in editing specific parts of the genome by removing, adding or altering sections of the DNA sequence

Although RAGE and its ligands are co-dependent in eliciting tumour concomitant cellular and molecular events, AGEs and RAGE can also independently facilitate tumour promotion via other non-receptor means and other ligands respectively, hence necessitating the simultaneous targeting of AGEs and RAGE duo for successful control of malignant cancers. Given that individual targeting of RAGE and its ligands have revealed sufficient inhibition of growth, spread and angiogenesis of tumours in multiple studies, the simultaneous targeting of the RAGE-ligand duo with RAGE-ligand antagonists should be strong enough to offer remarkable control over the “Achilles’ heel” of cancers, especially metastasis, therapy resistance, cancer resurgence and impaired patient prognosis [79]. Furthermore, the higher levels of (histone and MG) glycation adducts in tumour tissues than the normal counterparts, and also that of (MG) glycation adducts in advanced cancers than the localized ones [26, 48], reveal the diagnostic and prognostic implications of AGEs as potential bio-markers besides RAGE with deep clinical prospects.

As studied by Coluccio et al. [26], analyzing the BDCs of patients with cancer for the levels of glycation adducts could potentially serve to assess the stage and responsiveness of the disease, by deriving specific metabolomics profile of clinically exploitable molecular markers for developing precise and personalized treatment strategies. Attenuation of meta-inflammation and impaired insulin/IGF signalling by RAGE-targeted agents can be potentially exploited for optimal control of diabetes- and obesity-related cancers [77]. Impediment of RAGE-ligand signalling pathway has been shown to successfully mitigate the malignant behaviour of breast cancer cells [134]. El-far et al. [135], compiled the list of RAGE inhibitors that have shown promising effects in curbing cancer progression, including chondroitin sulphate, heparin sulphate, duloxetine, ergothioneine, ethyl pyruvate, hispidin, low molecular weight heparin and papaverine.

A subset of AGEs can be identified by means of their fluorescent characteristics. Recently, the cutaneous accretion of these fluorescent AGEs arising from long-lived hyperglycemia in diabetics were assessed by measuring SAF with an AGE reader, using excitation and emission range of 300–420 nm and 420–600 nm, respectively, spanning different AGEs. Type-2 diabetic patients who had cancer or went onto develop new cancers had considerably higher initial SAF values than those who did not have or develop cancer [47]. SAF values greater than 2.6 projected a 2.6 fold increased risk of cancer with significant concomitance between AGEs and cancer incidence (shown in Table 2). SAF is pertinent to pentosidine, an AGE prevalent in skin biopsies. Hence SAF, indicative of the AGEs accrued in skin, can be exploited as a non-invasive bio-marker for foreseeing the incidence of cancers in type-2 diabetic subjects. AGEs, as impending markers of glycemic memory, can be diagnostically utilised to predict cancer incidence in potentially susceptible individuals like diabetic patients.

Pouliquen and colleagues analysed various bio-markers from the existing literature data on invasive breast and colorectal cancer samples, including AGEs, AGE-modified proteins like annexin, prohibitin and fibrinogen, and RAGE for their significant pertinence and diagnostic relevance in invasive cancers [18]. Maksimovic and David [60] discuss novel strategies to unravel and analyse the NECM of histone like glycation, eliciting genomic instability in cancer, for its functional significance in diagnostic and prognostic purposes. Similarly, the blood cultures (liquid biopsy), serum/plasma, tissue samples (solid biopsy) and SAF (non-invasive) of patients with diabetes and/or cancer can be investigated for potentially risen levels of MG and other glycation adducts to monitor the disease stage and target treatment accordingly with AGE-RAGE inhibitors/antagonists in combination with conventional chemotherapy, providing prognostic values and potentiality of reverting or preventing cancer progression, metastasis and chemo-resistance.

Accretion of CML-AGE (Nε) was evidently concomitant with estrogen receptor expression, post-menopausal state and age of the cancer-afflicted patients, as determined by analyzing more than 200 mammary carcinoma samples. Furthermore, high CML-AGE accretion was substantially related to adverse prognosis and chemotherapy outcome in patients with estrogen-receptor negative (triple receptor negative and grade III carcinoma) breast cancer [22]. AGE accretion reflects the metabolic status of tumours, as cancer cells with high glycolytic rate undergoing Warburg effect accumulate high amounts of AGEs. As previously discussed in “Metabolic insurgence and cancer malignance” section, triple receptor negative breast cancers display aggressive progression, owing to their active Warburg’s type of glycolysis in both tumour cells and associated stromal components. Here, high CML-AGEs correlated with attenuated chemotherapeutic effects and aggressive behaviour of TNBCs, probably owing to their receptor (RAGE) activation eliciting chemo-resistance. With unlimited repercussions of AGE-invoked receptor RAGE signalling in cancer, AGEs can serve as potential biomarker for predicting cancer progression. Considering the pertinence of RAGE in creating a cancer-endorsing niche by up regulating hypoxia and inflammation, and instigating the progression of cancer stem cells, via its various ligands, including AGEs, HMGB1 and S100 proteins in TME, the implications of RAGE targeted therapeutic intervention for effective cancer control cannot be underestimated (Table 3) [89, 136, 137].

### AGE/RAGE-targeted nanoparticles in cancer diagnostics and therapeutics

Siddhartha and colleagues [114] devised DADS laden SLN conjugated with RAGE antibody (DADS-RAGE-SLN). Such nanoparticles surface-functionalized with RAGE were shown to exhibit enhanced bioavailability and augmented target specificity to cancer (TNBC/MDA-MB-231) cells, with elevated accretion in acidic tumour micro-milieu and increased (61.8%) cytotoxic impact on TNBC/MDA-MB-231 cells [114]. This acidic milieu-specific assembly of cytotoxic nanoparticles, owing to the conjugation with RAGE antibody, could incite tumour-specific toxicity with simultaneous evasion of non-selective toxicity to normal cells. Therefore, SLNs conjugated with RAGE could offer a potential therapeutic strategy for enhancing the anti-tumor efficacy of cytotoxic agents over cancer cells, without exerting undesirable effects on non-tumour cells.

Konopka et al. [116] designed a multi-modal imaging technique targeting RAGE, using pre-clinical studies and analysis of human samples, to aid in monitoring the diagnosis and prognosis of prostate cancer. Exploiting RAGE over-expression in cancer, they synthesized and confirmed the selectivity of multimodal nanoparticles effectively targeted at RAGE (^64^Cu-Cy5-G4-CML-detectable by nuclear and optical imaging), with potential implications in cancer management [116]. In an editorial of Theranostics, Drake and Scott [138] underlined the applicability of nanoparticle-based imaging agent conjugated with the ligand “CML-modified HSA” targeting RAGE, which was established by Konopka et al. [116]. They emphasise the significant pertinence of an innate ligand like CML-HSA (an advanced glycation end-product/AGE) conjugated nanoparticle to effectively target as well as image RAGE with successful theranostic implications [138].

Hence, we accentuate the multiple insinuations of both RAGE and its crucial ligand AGEs, in diagnostic as well as therapeutic aspects of cancer, owing to their pleiotropic role in initiation to multi-step invasion of cancers. While RAGE antibody-conjugated nanoparticles can bestow advantageous increase in bio-availability, cancer-specific cytotoxic efficacy and target specificity, AGE-conjugated nanoparticles can aid in targeting as well as imaging of RAGE over-expressing malignancies. This opens the ground for further investigation into the feasibility of novel “AGE-conjugated and RAGE-targeted” or “AGE-targeted and RAGE-conjugated” nanoparticles as well as other small molecule inhibitors-based theranostic interventions with impending prospects in early detection and efficient treatment of malignancies.

### RAGE-targeted nano-medicines in cancer therapeutics

ADCs are perfect models of dynamically targeted nano-medicines, consisting of a mAb coupled with cytotoxic drugs, which are then released into the cancer cells upon interaction of mAb with surface antigen, thereby inducing tumour cell death [121]. In 2019, Healey and colleagues [120] elaborated the development of a novel ADC against RAGE, suggesting it as a unique therapeutic target to successfully treat EC. The researchers analysed its efficacy, cellular uptake and intercellular transport *in vitro*, bio-distribution and toxicity *in vivo* in mice xenograft models. They revealed the augmented expression of RAGE in EC, adversely correlating with patient survival. RAGE-targeted ADCs were found to be up to 100-fold more effective in cancer cells than normal cells and 200-fold more cytotoxic compared to conventional drug administered individually. Moreover, RAGE-ADCs were proven to be non-toxic to normal mouse model, with substantial tumour growth suppression in mouse xenograft model of EC (shown in Table 3).

Healey and co-researchers [139] at Swansea University, Wales Cancer Research Centre, developed new RAGE-targeted ADCs and demonstrated their efficacy against ovarian and prostate cancers, which significantly express RAGE, yet lack efficient targeted drugs to treat. With platinum-based chemotherapy as the first-line of treatment at present, followed by distinct rounds of chemotherapy, ADCs against RAGE could be highly target-specific and potentially replace current chemo-treatment. Howard et al. [121], have studied the efficacy of RAGE-targeted ADCs against different gynaecological cancers. With pioneering researches and promising results in novel ADC-reliant treatments, ADCs form the largest class of nano-medicines being abundantly researched of late in pre-clinical and clinical trials for treating gynaecological cancers. Hence we propose that RAGE-targeted ADCs could play potential role as novel therapeutic agents with high efficacy and target specificity against not just gynaecological but also all RAGE over-expressing malignancies.

### Phyto-compounds as RAGE inhibitors

El‑Far et al. [124] recognised papaverine as a RAGE inhibitor by utilising the optimized‑peptide strategy of transformation to small molecules for drug designing. Papaverine elicited considerable suppression of RAGE-reliant cell proliferation, invasion and migration, RAGE-reliant NF-κB activation and RAGE expression in HT1080 human fibrosarcoma cells. Furthermore, papaverine-mimetic 3-styrylchromone derivative developed recently by Tanuma et al. [83] exhibited anti-inflammatory, anti-proliferative and anti-cancer effects via suppression of HMGB1-RAGE-ERK 1/2 signalling; anti-apoptotic effects via augmented pro-apoptotic Bax and caspase-3/7 expression in HCT116 colon cancer cells, both individually and in synergistic combination with DNA damaging agents. Hence we suggest that papaverine, scutellarein including many AGE inhibitors/anti-glycation agents (shown in Table 3) like curcumin, quercetin, withaferin A, and other plant-based agents could be developed as novel molecular lead compounds with inherent potential in anticancer therapeutics simultaneously non-toxic to healthy cells.

### Clinical trials evaluating the glycation triad—“AGEs, sRAGE and RAGE” as crucial bio-markers in cancer

Faruqui et al. [140] have emphasised the utilisation of RAGE inhibitors for targeted cancer treatment. They have different RAGE inhibitors varying from as simple non-specific drug as metformin and heparin, to highly specific RAGE inhibitors like FPS-ZM1, which all require further clinical trials to evaluate their efficacy [140]. We have previously listed different possible lead compounds targeting RAGE and its ligands—AGEs, HMGB1 and S100 group of proteins, ranging from pre-existing drugs (used to treat other conditions) like metformin, calcimycin, cromolyn, pentamidine, sulindac, tasquinimod and methotrexate to RAGE-specific inhibitors like antibodies, aptamers and peptibodies to RAGE or its ligands [12]. They all have the potential to be translated as effective anti-cancer drugs, but only made possible by detailed assessment through clinical trials.

Oral vitamin D3 (50,000 IU), administered once every 8 weeks, was found to augment sRAGE levels in women with PCOS, as reported in a recent phase IV clinical trial conducted by Maimonides Medical Center, USA [130]. Now this result is highly crucial with respect to the fact that reduced sRAGE levels and high plasma AGEs have been reported in patients with breast cancer [41]. In this most recent study conducted in Tianjin Medical University Cancer Institute and Hospital, China, decreased sRAGE and elevated AGE levels were found to increase breast cancer risk, thereby establishing negative concomitance of sRAGE and positive association of AGEs, respectively, with breast cancer. Hence vitamin D might be exploited to reduce sRAGE levels in cancer-susceptible and -affected patients with preventive and therapeutic implications. Vitamin D has been employed in clinical trials for analyzing its efficacy in suppression of various inflammatory markers including AGEs, sRAGE and RAGE, in combination with plant-based compounds like grape seed extract, in patients with advanced cancer [129]. Resveratrol, a polyphenol present in grape seed extract might be responsible for its anti-inflammatory and anti-glycation activities, with potential for RAGE inhibition in cancer cells [141]. Ongoing clinical trial in a Turkish University evaluates the relationship between dietary AGE consumption, inflammation and oxidative stress in breast cancer patients [132]. A recent clinical trial involving dietary intervention low in AGEs, with predictive implications in breast cancer survivors, to reduce known (CRP and IL-6) and novel (AGE and RAGE) prognostic biomarkers, has been conducted in Washington University School of Medicine (WUSOM) [133]. Another clinical trial (phase I) carried out by WUSOM investigated the rise in cytotoxic effects of azacitidine when combined with CX-01, the inhibitor of HMGB1-RAGE/TLR4 interactions and chemokine receptor CXCR4 axis, on MDS and AML [128].

Phase I and II clinical trials of an oral RAGE inhibitor azeliragon, with insinuations in attenuating inflammation and hence side effects of chemotherapy, including anthracycline-induced cardiotoxicity and chemotherapy-induced cognitive debility in patients with breast cancer, and cerebral edema post-surgery in patients with glioblastoma, via inhibition of RAGE pathway have been conducted most recently in Georgetown University, Washington and City of Hope Medical Center, California, respectively [125, 127]. Besides potential exploitation in reducing the undesirable impact of chemotherapy, this RAGE inhibitor is also used to achieve dose reduction of drugs by combined usage with main drugs like dexamethasone employed to subside cerebral edema ensuing surgery in patients with glioblastoma. Further phase I and II clinical trials have been conducted by Cedars-Sinai Medical Center, California, with azeliragon to treat metastatic pancreatic cancer, which exhibits resistance to first-line of chemotherapy [126]. Hence multiple research studies including clinical trials (as shown in Tables 2 and 3) are underway to unravel the substantial concomitance of glycation triad—AGEs, sRAGE and RAGE, as potent diagnostic and therapeutic biomarkers with translational significance in cancer. Furthermore, inhibitors of AGE-RAGE axis like DPP-IV inhibitors (sitagliptin) have also been tested in other impaired metabolic conditions like type-2 diabetes to possibly work as a cancer-protective agent in diabetes by disrupting the AGE-RAGE cues [131].

## Conclusions

The inception of late undesirable aftermaths of predominantly high plasma AGEs in the cancer recovered subjects, mortality of cancer-afflicted patients from high dietary AGEs and incidence of cancers in the diabetic patients with high cutaneous AGEs than in the healthy population, are evidently pertinent to the dysregulated AGE signalling plus activated RAGE pathway. Implication of both exogenous and endogenous AGEs in the pre-cancer risk and incidence, cancer progression and mortality, and post-cancer complications, necessitate the advent of drugs capable of inhibiting the formation of total AGEs and AGE-activated RAGE, as combination agents in cancer chemotherapy for the plausible enhanced advantage. Moreover, RAGE inhibitors like azeliragon and FPS-ZM1, nano-medicines and nano-particles targeting AGE-RAGE axis, and natural polyphenols inhibiting AGEs, in combination with synbiotics, all need to be evaluated further in clinical trials to be employed as combination agents with conventional chemotherapy for cancer. Utilising different AGE-RAGE inhibitors eliciting pleiotropic effects, in combination with clinical cytotoxic drugs can aid in evading metastasis, chemo-resistance and cancer resurgence, the most challenging aspects of cancer treatment as of now. Newer studies on the molecular components regulated by AGEs like epigenome, gut microbiome and microbial metabolome, underline the diverse and universal role of AGEs in promoting cancer malignancy via metastasis. Dietary AGEs also need to be restricted and watched for, considering their immense interplay between normal healthy and cancerous milieu. The irreversible “metabolic imprint” of AGEs instigating molecular signature of RAGE, giving rise to metastatic or malignant cancers cannot be under-estimated, and need to be further studied for possible intervention with natural anti-glycative polyphenols and synbiotics as food additives or chemo-preventive agents. All these findings accentuate the sheer requisite of anti-glycation compounds/AGE/RAGE antagonists as potentially active components of successful cancer treatment, considering the multiplicity of AGEs with vital role in inducing RAGE and its other ligands. Due to its affinity for multiple ligands and its influence on multi-stage cancer promoting effects (proliferation, invasion, metastasis and angiogenesis), RAGE serves as an indispensable bio-marker in evaluating the prognosis of different cancers, besides being a novel lucrative target for precise and personalized treatment of cancers, based on the patient’s individual expression levels. Furthermore, many studies are required to unravel the diagnostic and therapeutic impact of this AGE-RAGE duo as markers, considering their impending clinical implications.

## Abbreviations

ADC: antibody-drug conjugate
AGE: advanced glycation end product
AKT: protein kinase B
AML: acute myeloid leukemia
AP-1: activating protein-1
Bax: B cell lymphoma protein-2 associated X protein
Bcl-2: B cell lymphoma protein-2
BDCs: blood-derived cultures
CAFs: cancer-associated fibroblasts
c-Fos: FBJ murine osteosarcoma viral oncogene homolog
CML: carboxy-methyl lysine
CRP: c-reactive protein
CXCR4: C-X-C chemokine receptor 4
Cy5: Cyanine5 dye
DADS: diallyl-disulphide
DAMPs: damage-associated molecular patterns
DJ-1: deglycase enzyme
DPP-IV: dipeptidyl peptidase 4
EC: endometrial cancer
ECM: extracellular matrix
EMT: epithelial-mesenchymal transition
ERK 1/2: extracellular signal-regulated kinase 1/2
GLO-1: glyoxalase-1
GLUT-1: glucose transporter-1
GSK-3β: glycogen synthase kinase 3β
HDL: high-density lipoprotein
HIF-1α: hypoxia-inducible factor 1α
HL: Hodgkin’s lymphoma
HMGB1: high mobility group box-1
HSA: human serum albumin
HSPs: heat shock proteins
IGF-1: insulin-like growth factor 1
IL-6: interleukin-6
IκB: nuclear factor kappa B inhibitor
LC-3: microtubule-associated protein light chain 3
LPS: lipopolysaccharide
mAb: monoclonal antibody
MAC-1: macrophage-1
MAPK: mitogen-activated protein kinase
MCT-4: monocarboxylate transporter-4
MDS: myelo-dysplastic syndrome
MDSC: myeloid-derived suppressor cell
MG: methylglyoxal
MGAs: methylglyoxal adducts
MG-H1: methylglyoxal-derived hydroimidazolone
MHC: major histocompatibility complex
MMP1: matrix metalloproteinase 1
mRNA: messenger RNA
mTOR: mammalian target of rapamycin
NADPH: reduced nicotinamide adenine dinucleotide phosphate
NECM: non-enzymatic covalent modifications
NF-κB: nuclear factor kappa B
NOX-2: reduced nicotinamide adenine dinucleotide phosphate oxidase enzyme-2
NRF-2: nuclear factor erythroid 2-related factor 2
PCOS: polycystic ovary syndrome
PI3K: phosphatidylinositol 3-kinase
PRR: pattern recognition receptor
RAGE: receptor for advanced glycation end product
Ras: rat sarcoma protein
RNAi: interfering RNA
ROS: reactive oxygen species
SAF: skin auto-fluorescence
SLN: solid-lipid nanoparticle
SP-1: stimulator protein-1
sRAGE: soluble receptor for advanced glycation end product
TAM: tumor associated macrophage
TGF-β: transforming growth factor β
TLR4: Toll-like receptor 4
TME: tumour micro-environment
TNBC: triple negative breast cancer
TNF: tumour necrosis factor
VEGF: vascular endothelial growth factor

## Declarations

### Copyright

© The Author(s) 2023.
